# A Bibliometric Analysis of Strategies for Atherosclerosis Treatment with Organic Nanoparticles

**DOI:** 10.3390/pharmaceutics17091131

**Published:** 2025-08-29

**Authors:** Jizhuang Ma, Xia Zhao, Xinwen Xu, Lixin A, Qiang Liu, Peng Qu

**Affiliations:** Faculty of Medicine, Dalian University of Technology, Dalian 116024, China

**Keywords:** atherosclerosis, bibliometric analysis, drug delivery, organic nanoparticles, nanotechnology

## Abstract

The complex pathological mechanisms of atherosclerosis (AS) involve lipid metabolism disorders, inflammatory responses, and plaque instability, resulting in significant challenges to effective clinical management. Current therapeutic approaches, such as statins and stent implantation, suffer from issues including single-target action, notable side effects, and the risk of restenosis. Nanoparticle-based drug delivery systems have demonstrated considerable promise by enabling the codelivery of multiple agents directly to atherosclerotic lesions, thereby improving therapeutic efficacy and minimizing systemic toxicity. Among various nanomaterials, organic nanoparticles have recently emerged as a research hotspot in the field of AS treatment due to their excellent biocompatibility, degradability, and potential for targeted modification. This review systematically summarizes the recent advances and emerging trends in the application of organic nanoparticles for AS treatment, employing bibliometric analysis to delineate research frontiers. We employed bibliometric tools to analyze 1999 articles on organic nanocarriers for AS therapy indexed in the Web of Science Core Collection. The analysis included co-occurrence and clustering techniques to explore influential keywords and key contributors. Temporal analysis was applied to identify emerging research hotspots and track the evolution of this field. The literature reveals three major current focal areas: (1) the development of engineered biomimetic organic nanoparticles; (2) the design of multifunctional polymer-based organic nanocarriers; and (3) the innovation of organic-coated stents. This article not only provides a comprehensive overview of cutting-edge organic nanotechnologies for AS therapy, but also critically discusses the challenges in clinical translation, offering insights into future directions for the development of safe, effective, and personalized nanomedicine strategies against AS.

## 1. Introduction

Atherosclerosis (AS) is a chronic disease characterized by lipid deposition in the vascular wall, inflammatory responses, and fibrous tissue hyperplasia, serving as the primary pathological basis of cardiovascular diseases worldwide [1]. According to the World Health Organization (WHO), AS and its complications are responsible for over 17 million deaths annually, accounting for approximately 31% of global mortality [2]. Aortic AS may trigger aortic dissection or aneurysm, with a rupture risk leading to a mortality rate exceeding 50% [3]. The pathogenesis of AS involves the interaction of multiple factors, with recognized theories including the endothelial injury response hypothesis, lipid infiltration hypothesis, and chronic inflammation hypothesis [4]. Among these, vascular endothelial dysfunction is considered the initiating event of AS, while lipid metabolism disorders and inflammation responses are critical contributors throughout disease progression [5]. Vascular endothelial cells act as a critical barrier for maintaining vascular homeostasis [6]. Risk factors such as hypertension, hyperlipidemia, smoking, diabetes, and oxidative stress can damage endothelial cells, leading to functional abnormalities [7]. Impaired endothelial cells exhibit increased permeability, allowing low-density lipoprotein (LDL) to enter the intima, where it undergoes local oxidation to form oxidized LDL (ox-LDL). ox-LDL further induces endothelial cells to express adhesion molecules and chemokines, attracting monocytes to migrate from the bloodstream into the subintima [8]. Monocytes differentiating into macrophages within the intima excessively uptake ox-LDL via scavenger receptors, leading to intracellular lipid accumulation and formation of foam cells [9]. The accumulation of foam cells is a typical feature of the early AS lesion, such as the “lipid streak”. As the lesion progresses, foam cells gradually die, releasing lipids and pro-inflammatory factors to form a lipid core [10]. Stimulated by the inflammatory microenvironment, vascular smooth muscle cells (VSMCs) migrate into the intima and proliferate, secreting abundant extracellular matrix to form a fibrous cap [11]. The composition and stability of this fibrous cap are critical in determining plaque vulnerability [12]. However, enzymatic degradation by matrix metalloproteinases (MMPs) and persistent inflammation may weaken the cap, increasing the risk of rupture and thrombotic events [13]. These complex pathophysiological mechanisms render AS highly challenging to treat. Current clinical treatments include drug therapy and interventional therapy, both with significant limitations [14]. Statins, widely used as lipid-lowering drugs, inhibit hydroxymethylglutaryl-coenzyme A (HMG-CoA) reductase to reduce cholesterol synthesis [15]. However, long-term high-dose use may cause side effects such as liver injury and myotoxicity [16,17]. Probucol exhibits antioxidant and anti-inflammatory effects but is limited by low solubility and bioavailability [18]. Gene drugs, despite their low toxicity and high efficacy, are prone to degradation in the bloodstream, restricting their application [19]. Stent implantation, a key intervention for AS-related vascular stenosis, faces challenges such as in-stent restenosis and thrombosis. Vascular wall injury following stent implantation triggers inflammation and smooth muscle cell proliferation, leading to restenosis [20]. Promising progress in AS treatment has been achieved through targeted drug delivery using nanoparticles and the development of drug-eluting stents [21,22]. In recent years, nanotechnology-based drug delivery systems have emerged as promising strategies to overcome these limitations.

Nanoparticles, ranging from 1 to 1000 nm, can encapsulate, adsorb, or conjugate therapeutic agents including chemical drugs, proteins and nucleic acids, which enable controlled release, precise delivery and enhanced efficacy [23,24]. Based on composition, nanoparticles are broadly categorized into inorganic and organic types [25]. While a few inorganic nanoparticles, such as iron oxide and silica-gold nanoparticles, have entered clinical trials for AS treatment, most require extensive safety evaluations (such as long-term accumulation of toxicity in the body) for clinical translation [26]. In contrast, organic nanoparticles such as liposomes, micelles and polymer-based carriers have been demonstrated superior biocompatibility, biodegradability and clinical translatability [27]. Materials such as phospholipids and hyaluronic acid are endogenous or naturally derived, while synthetic polymers like PLA and PLGA degrade into metabolizable components [28]. Moreover, biomimetic organic nanoparticles such as extracellular vesicles, possess immune-evasive properties and enhanced targeting capabilities, further improving therapeutic outcomes [29].

Bibliometrics relies on mathematical and statistical methods to sort out and analyze scientific research activities in specific research fields. Based on literature databases and metric attributes, it further identifies research frontiers and hot directions, providing strong strategic support for research. With the help of bibliometric analysis, researchers can easily grasp the publication dynamics of a specific research field in terms of annual publication growth, most influential countries/institutions, literature sources, and research hotspots, thereby forming an overall understanding of the field’s influence and development trends. In addition, bibliometric analysis can also objectively analyze the current status of a specific field and provide guidance for its future development path.

**Scheme 1:**
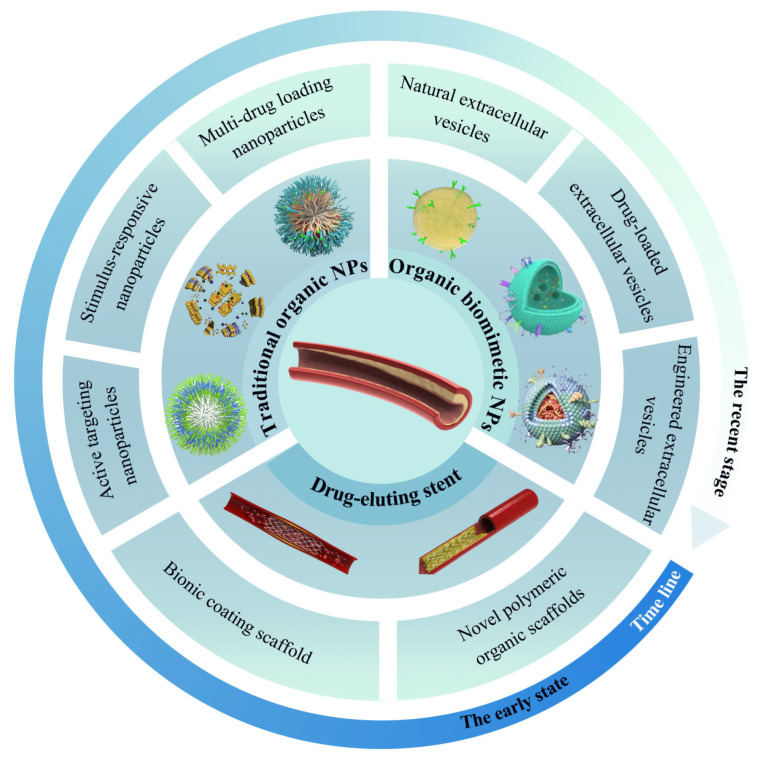
The main research hotspots and time evolution of the application of organic nanoparticles for AS treatment.

Given the growing number of studies and rapid advancements in the field. This review employs bibliometric analysis to systematically examine recent research trends and identify hotspots in the application of organic nanoparticles for atherosclerosis treatment. Compared to previous reviews that primarily focused on inorganic nanocarriers, this work aims to highlight the innovative potential, translational advantages, and future directions of organic nanomaterials in AS therapy (Scheme 1).

## 2. Bibliometric Analysis

### 2.1. Data Collection

Our review is based on the collected data from a series of research results in queries to the Web of Science (WoS) core collection and the Editions is Science Citation Index Expanded (SCI-EXPANDED)—1945-present. Web of Science (WOS) is widely regarded by researchers as a high-quality digital literature database and is also recognized as the optimal choice for bibliometric analysis. As a comprehensive multidisciplinary database, it covers all high-impact scientific journals and world-class indexing resources. Compared with databases such as Scopus or MEDLINE/PubMed, WOS can extract more complete information for bibliometric analysis. However, this database still has certain limitations, such as incomplete coverage and characterization of literature, as well as issues like language bias, time bias, and subject bias. First, thorough key search terms: TS = (atherosclero*), limit the disease to atherosclerosis. Second, our review aims to display the application of organic nanoparticles for atherosclerosis treatment comprehensively, hence, the second query focuses on the object of research domains: TS = (liposome* OR micelle* OR dendrimer* OR polymer OR “polymeric nanoparticle*” OR nanocapsule* OR hydrogel* OR “extracellular vesicle*” OR “protein cage*” OR exosome* OR “solid lipid nanoparticle*” OR “nanostructured lipid carrier*” OR “lipid-polymer hybrid NP*” OR “DNA block copolymer*” OR “polysaccharide nanoparticle” OR “chitosan nanoparticle” OR “hyaluronic acid nanoparticle*” OR “albumin nanoparticle” OR “protein-based nanoparticle” OR “cyclodextrin complex” OR “inclusion complex” OR “nanoemulsion” OR “microemulsion” OR “polymersome” OR “biomimetic nanoparticle” OR “high-density lipoprotein nanoparticles” OR “carbon nanotubes”, “graphene” and “fullerenes”). Finally, the document type is restricted to the article.

### 2.2. Visualization and Analysis

Following data acquisition, the collected information underwent processing and graphical representation via the native analytical tools of Web of Science (WoS) and the scientometric visualization software VOSviewer (version 1.6.17, developed by Leiden University’s Centre for Science and Technology Studies). WoS facilitated dataset extraction and preliminary statistical evaluations, including chronological publication trends and geographic distribution of contributing regions. VOSviewer, a specialized platform for scientometric exploration, enabled the mapping of thematic clusters and the identification of evolving patterns through co-occurrence network analysis and density-based visualization techniques.

#### 2.2.1. Publications Analysis

**Figure 1:**
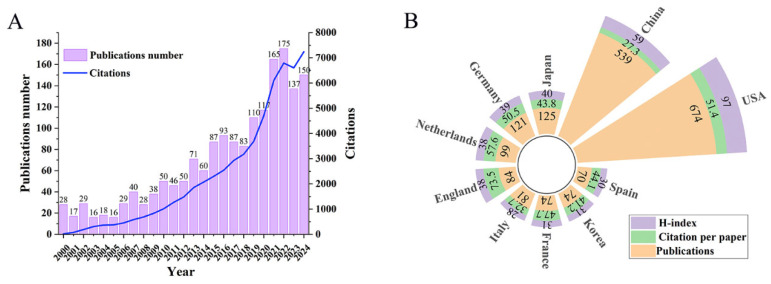
(**A**) The annual publication and citation growth of the literature regarding the application of organic nanoparticles for AS treatment. (**B**) The most contributing countries in the application of organic nanoparticles for AS treatment.

A total of 1998 articles related to the application of organic nanoparticles for AS treatment were retrieved from the Web of Science (WoS) Core Collection. The annual publication count, which reflects the developmental trends in the related research field, showed that from 2000 to 2018, the annual number of WOS core colletion publications remained below 100. Since 2019, however, annual WOS core colletion publications have consistently exceeded 100, accompanied by a continuous increase in citation counts (Figure 1A). The growth in citations suggests that research on the application of organic nanoparticles for AS treatment is gaining recognition, and it is likely to be an area of continued growth and interest in the future. Further analysis of research contributors (Figure 1B) revealed that the top ten countries by publication output are the United States (674), China (539), Japan (125), Germany (121), North Island (99), the United Kingdom (84), Italy (81), France (74), South Korea (74), and Spain (70). Among these, the United States exhibited the highest H-index for related publications, indicating that it has produced a significant number of highly cited papers. The research on the application of organic nanoparticles for AS is driven mostly by America and Europe.

#### 2.2.2. Major Research Domains

To deeply explore the classification and research priorities of the organic nanoparticles used for AS treatment, this study conducts a systematic analysis through keyword co-occurrence analysis and in-depth data mining. The keyword co-occurrence network is constructed based on the number of studies where two keywords appear simultaneously in titles, abstracts, or keyword lists, with author keywords effectively characterizing research focuses and trends. After setting a minimum occurrence frequency of 15, 217 keywords were extracted from 1998 studies, mainly forming three color-coded clusters (Figure S1A), each representing a specific research direction. In addition to the core keyword “atherosclerosis”, the high-frequency keywords include “inflammation” (190 times), “extracellular carriers” (174 times), “nanoparticles” and “nanoparticle” (128 times), “macrophages” and “macrophage” (108 times), “exosomes” and “exosome” (99 times). Cluster analysis reveals three main groups. The green cluster, centered on “inflammation”, “extracellular vesicles”, and “exosomes”, covers the application of organic biomimetic nanocarriers in AS treatment. The red cluster focuses on drug delivery using traditional organic nanoparticles, with “nanoparticles”, “macrophages” and “drug-delivery” being the most prominent terms. The blue cluster is dedicated to stent materials in AS therapy, with “restenosis”, “drug-eluting stents” and “polymers” being the most prominent terms. By comprehensively analyzing the internal relationships among these clusters, the organic nanoparticles used for AS treatment can be categorized into three major research domains: organic biomimetic nanocarriers, traditional organic nanocarriers, and organic stent materials.

The temporal distribution of research terms effectively reflects the dynamic evolution of the field. Therefore, this study further analyzes the evolutionary trajectory of keywords through a time-based dimension to reveal the development trends in the organic nanoparticles used for AS treatment (Figure S1B). In the constructed time-evolution map (node color coding corresponds to the temporal dimension of research hotspots), dark purple nodes represent traditional research directions emerging in 2014 or earlier, while bright yellow nodes mark emerging fields that have emerged in the past five years. Temporal analysis shows that stent materials (such as polymers), as the core of the dark purple cluster, confirm the critical role of this technology in AS treatment through their early dominant position. As time passed, traditional nanocarriers (such as polymeric nanoparticles) in the green cluster have gradually become a research hotspot since 2016. Notably, organic biomimetic nanocarriers (such as extracellular vesicles and exosomes) within the yellow cluster have become current research hotspots.

## 3. Research Hotspots of Organic Nanoparticles for as Treatment

### 3.1. Organic Coated Stents

As shown in the bibliometric analysis, the blue cluster in the Figure S1A highlights “restenosis” and “stent materials” as high-frequency keywords, whose temporal distribution indicates that systematic research in this field was established in the early stages. After 40 years of development, vascular stents have been widely used in the interventional treatment of coronary heart disease, particularly percutaneous coronary intervention (PCI), which has significantly reduced the mortality rate of acute myocardial infarction by relieving atherosclerotic obstruction of the coronary arteries [30]. However, the first-generation bare metal stents (BMS) were found to cause late postoperative complications in some patients during clinical application, including late stent thrombosis (LST), in-stent restenosis (ISR), and in-stent neoatherosclerosis (ISNA) [31]. These complications can trigger acute coronary syndromes, manifesting as angina pectoris, myocardial infarction, and even sudden cardiac death. The second-generation drug-eluting stents (DES) use non-degradable polymers as drug carriers, with drug release relying on passive diffusion mechanisms. However, long-term retention of non-degradable polymers continuously stimulates vascular tissues, inducing chronic inflammatory responses [32]. The third-generation DES has been improved with biodegradable polymers, enabling programmed drug release during the degradation process [33]. Although the third-generation stents have significantly improved therapeutic effects, issues such as postoperative inflammatory responses and drug coatings inhibiting vascular endothelial cell proliferation have not been fully resolved [34]. To overcome these technical bottlenecks, the precise design of DES from a materials science perspective is required, including the development of new polymer material coatings and the loading of new drugs.

#### 3.1.1. New Polymeric Organics as Stent Coatings

**Figure 2:**
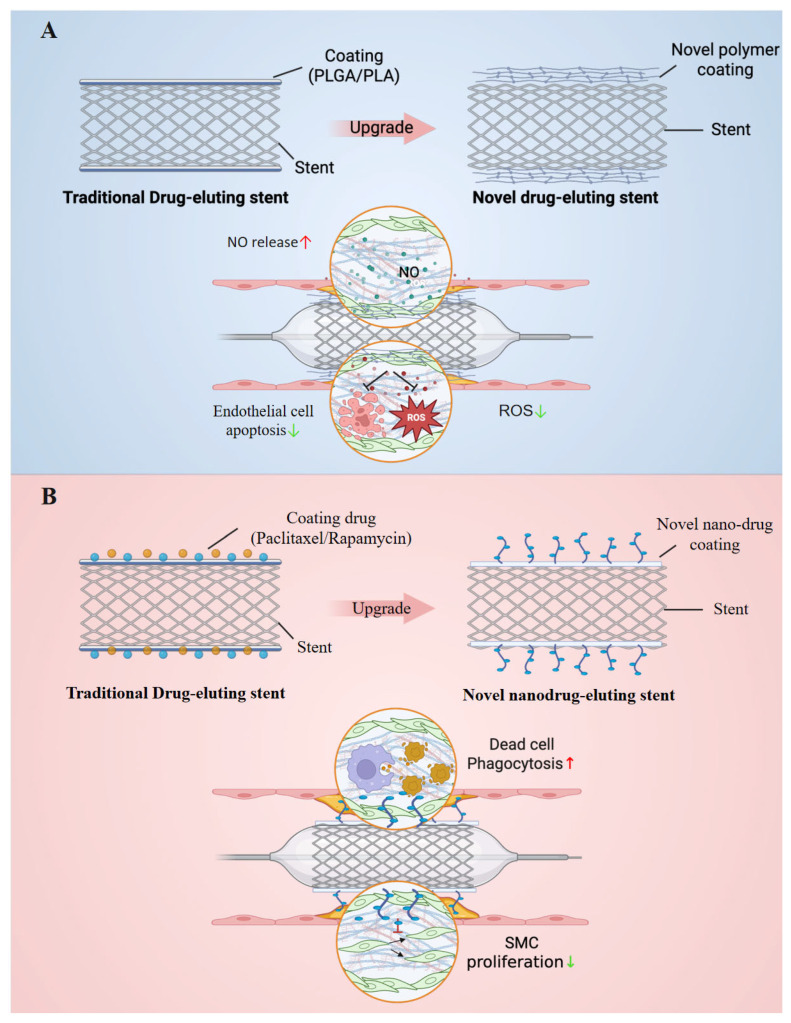
Novel drug-eluting stents designed for the treatment of atherosclerosis. (**A**) The novel polymer-coated scaffold has antioxidant and nitric oxide release functions, replacing the traditional PLGA/PLA coating. (**B**) The novel scaffold with nano-drug coating can enhance the clearance of apoptotic cells and inhibit the proliferation of smooth muscle cells, replacing the traditional drug-coated scaffolds containing paclitaxel and rapamycin. The red arrow represents enhancement, and the green arrow represents inhibition.

The third-generation DES, such as the Orsiro and SYNERGY stent, has mitigated in-stent restenosis through material innovation. The Orsiro stent uses a polylactic acid (PLA) degradable polymer drug delivery system loaded with rapamycin for programmed release, achieving 80% drug release within 3 months, with complete degradation of the polymer coating within 12–24 months [35]. The SYNERGY stent innovatively applies a 4 μm ultra-thin poly(lactic-co-glycolic acid) (PLGA) coating that only covers the lumen side of the stent to reduce polymer exposure. Pharmacokinetic studies have confirmed that everolimus is 80% released within 90 days, and the PLGA matrix degrades within 4 months [36]. Despite the progress of third-generation DES, early foreign body reactions remain a clinical challenge. Within 24 h after stent implantation, mechanical dilation disrupts the integrity of the endothelial cell (EC) layer, and the acidic microenvironment (pH 5.8–6.2) generated by PLGA/PLA degradation continues to stimulate, potentially exacerbating the intensity of the inflammatory response [37]. Therefore, endothelial dysfunction and chronic inflammation remain challenges in the clinical application of modern stent technology, which prompts researchers to develop new polymer-coated materials (Figure 2A).

##### Vascular Reendothelialization Coated Stents

Endothelial dysfunction refers to structural or functional abnormalities of vascular endothelial cells, leading to impaired regulation of vascular homeostasis, manifested as pathological states such as vasomotor dysfunction, imbalance between anti-inflammatory and pro-inflammatory responses, and disorders of coagulation/anticoagulation [38]. In the development of functional coatings for vascular stents, biomimetic strategies and the regulation of endogenous gas molecules have become hotspots in recent years. Nitric oxide (NO) plays an indispensable role in cardiovascular health by regulating vascular tension, inhibiting platelet aggregation, and reducing the proliferation of vascular smooth muscle. NO donors include organic nitrates, S-nitroso mercaptans, NONOates, metal-NO complexes, etc. NO can be released through enzymes, light, pH changes or spontaneous triggering. However, these materials are confronted with challenges such as large-scale production and long-term stability. Therefore, it is necessary to develop methods to stimulate the endogenous production of nitric oxide. S-nitrosothiols is a stable carrier of NO, transporting NO to target tissues in the blood to prevent rapid oxidation and failure of NO. Glutathione peroxidase (GPx) is a family of antioxidant enzymes containing selenocysteine, which releases functional NO by decomposing S-nitrosothiols. Yang et al. [39] developed a catalytic NO coating with endothelial biomimetic functions. A polyallylamine (PPAam) substrate layer was constructed via plasma polymerization, covalently bound to 3,3-diselenodipropionic acid (SeDPA) with GPx activity, enabling continuous catalytic release of NO from endogenous S-nitrosothiols. This coating inhibits human umbilical artery smooth muscle cell proliferation by activating the cGMP signaling pathway, with an inhibition rate >60%. Notably, the coating has a bidirectional regulatory effect. While inhibiting smooth muscle cell migration, it promotes human umbilical vein endothelial cell migration. Animal experiments confirmed that the coated stent significantly promoted vascular reendothelialization after 28 days, with an endothelial coverage rate of 90%. Similarly, Zhang et al. [40] developed a biomimetic nanozyme coating, where zinc ions (Zn^2+^) and lysozyme self-assemble to form a nanoparticle composite coating BMC. This design mimics the activity of GPx, enabling sustained catalytic release of NO. In a rabbit abdominal aorta model, the endothelial coverage rate increased to 2.3 times that of the control group at 4 weeks, and intimal thickness decreased by 65% at 24 weeks, providing new insights for the design of multifunctional vascular stents.

##### Anti-Inflammation Coated Stents

Vascular mechanical injury caused by stent implantation activates neutrophils and macrophages, releasing large amounts of ROS (such as O_2_^−^, H_2_O_2_), increasing local ROS levels [41]. This oxidative stress environment significantly promotes in-stent restenosis (ISR) by activating pro-inflammatory signaling pathways and inducing abnormal proliferation of vascular smooth muscle cells [42]. However, the PLA/PLGA coatings used in third-generation DES lack ROS-scavenging capabilities and cannot effectively block this pathological process. To address inflammation caused by drug-coated stents, researchers have developed new polymeric coating materials with anti-inflammatory effects. Van Lith et al. [43] directly covalently polymerized drug molecules to create polymeric materials. By copolymerizing ascorbic acid (AA) with citric acid, they designed a degradable polyester elastomer (POCA) with both free radical scavenging and metal chelating abilities. POCA regulates degradation rate through ester bond polymerization, and its active components continuously scavenge ROS with an efficiency of 85%. In a guinea pig aortic transplant model, POCA-coated stents exhibited significantly reduced intimal hyperplasia, with histological analysis showing a ~35% reduction in neointimal thickness, and degradation products of POCA retaining antioxidant activity. Recently, Liu et al. [44] investigated hyaluronic acid (HA), a polymer abundant in the human body, as a stent coating material, constructing a baicalin-hyaluronic acid (BCL-HA) composite functional coating. In this design, BCL inhibits hyaluronidase activity and scavenges ROS (H_2_O_2_ consumption reached 25 nmol/cm^2^), effectively protecting HA from degradation. Animal models showed it maintained vascular patency and reduced intimal hyperplasia. This dual-protection strategy based on natural products provides a new solution for improving long-term stent stability.

#### 3.1.2. Biomimetic Nanoparticles as Stent Drugs

DES typically uses chemical drugs such as paclitaxel or rapamycin [45]. Paclitaxel stabilizes tubulin structure, prevents microtubule depolymerization required for cell division, and arrests smooth muscle cells in the G0/G1 or G2/M phase. Its high lipophilicity allows rapid penetration of the vascular wall, forming a high-concentration drug pool locally and prolonging its action. Compared with paclitaxel, rapamycin has a smaller molecular weight (~914 Da) and better tissue penetration. Rapamycin binds to FKBP12 protein to form a complex, blocking the mTOR signaling pathway, inhibiting smooth muscle cell proliferation and migration, while delaying endothelialization [46]. Its anti-inflammatory effect reduces local inflammation after stent implantation and lowers thrombosis risk. Although paclitaxel and rapamycin induce apoptosis of smooth muscle cells, they also suppress endothelial cell proliferation [34]. Additionally, defective efferocytosis of macrophages in atherosclerotic plaques after stent implantation leads to impaired clearance of drug-induced apoptotic cells, triggering persistent inflammation and neointimal hyperplasia [47]. To address the above issues, DES drugs were replaced with less toxic therapeutic drugs, such as genes and proteins [48,49]. Nanoparticles such as liposomes and chitosan are used to target and deliver these genes or proteins to smooth muscle cells [50]. Recently, biomimetic nanoparticles have shown great advantages in AS treatment, and strategies combining biomimetic nanoparticles with stents are being widely researched (Figure 2B). Researchers have prepared novel biomimetic stents, such as in vitro endothelialized stents, stem cell-delivery stents, and extracellular vesicle stents. For example, Zou et al. [51] developed a lipoprotein-associated phospholipase A2 (Lp-PLA2)-responsive multivesicular stent coating loaded with mesenchymal stem cell (MSC) exosomes to prevent in-stent restenosis. The study found MSC exosomes enhance macrophage efferocytosis via SLC2a1/STAT3/RAC1 and CD300a pathways, inhibit foam cell formation by downregulating CD36, and their miR-143 suppresses smooth muscle phenotypic transformation to synergistically inhibit neointimal hyperplasia. Animal study confirmed significant reduction in neointimal thickness and improvement of the inflammatory microenvironment. This study first integrated efferocytosis repair mechanisms into stent design, providing a paradigm for targeted intervention in complex pathological microenvironments. Another study constructed heparinized polycaprolactone stents loaded with MSC exosomes (PCL-Hep/sEV) [52]. Results showed that exosomes promoted endothelialization by delivering active components such as VEGF/miR-126, increasing CD31+ coverage by 40%, and regulating macrophage M2 polarization, decreasing the M1/M2 ratio by 60%, thereby inhibiting inflammation-mediated calcification. Notably, the stent successfully overcame the calcification risk caused by pure heparin coatings, with a 67% reduction in calcification volume, confirming the dual functions of exosomes in balancing anticoagulation and tissue regeneration. In general, replacing the traditional chemical drug paclitaxel with EVs from natural sources as therapeutic drugs has the therapeutic effect of enhancing efficacy and reducing toxicity.

### 3.2. Traditional Organic Polymeric Nanoparticles

In the hotspot areas identified by bibliometric analysis (Figure S1A), the red cluster indicates that the application of “nanoparticles” (particularly liposomes and polymeric materials) in AS treatment is a high-frequency term. The advantages of traditional nanoparticles include sustained drug release, easy targeting design, the ability to construct intelligent responsive systems, and the capacity to load multiple drugs for synergistic therapy [53]. Loading drugs with organic nanoparticles can increase drug concentration at the lesion site and reduce drug exposure in other tissues, thereby enhancing therapeutic effects and minimizing adverse reactions. Further classify the relevant literature, the design of organic polymeric nanoparticles for AS therapy can be mainly categorized into three main types: targeted nanoparticles, stimulus-responsive nanoparticles, and multifunctional synergistic nanoparticles.

#### 3.2.1. Targeted Nanoparticles

**Figure 3:**
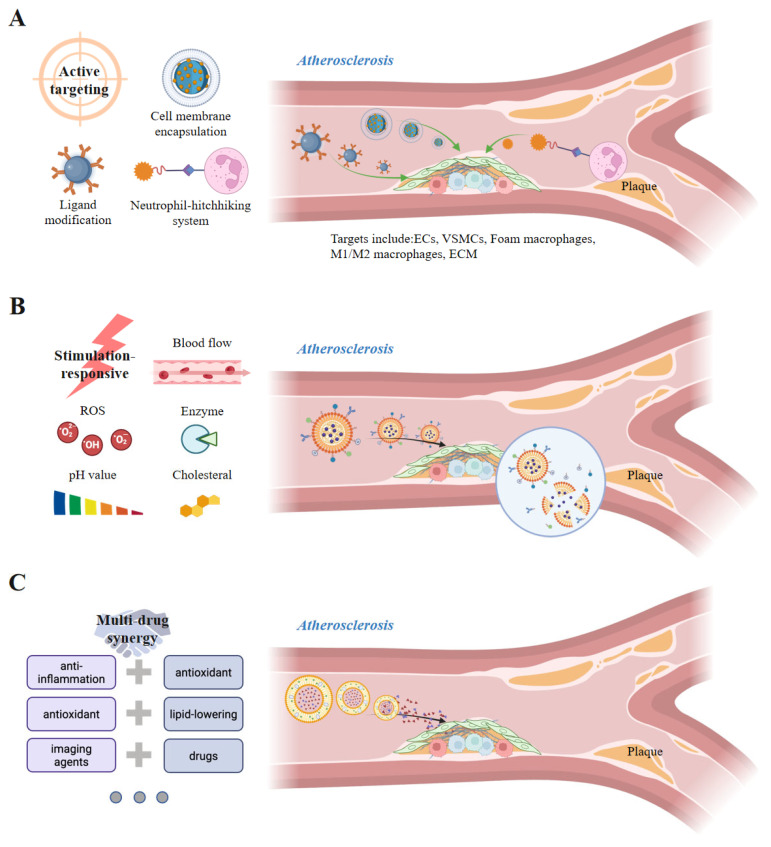
Design of traditional organic nanocarriers for the treatment of atherosclerosis. (**A**) Targeted nanocarriers for the treatment of atherosclerosis, including ligand modification, cell membrane encapsulation, and neutrophil hijacking. The targets include ECs, VSMCs, Foam macrophages, M1/M2 macrophages, ECM, etc. (**B**) Intelligent responsive nanocarriers for the treatment of atherosclerosis. The stimulation methods include ROS, pH, blood flow, enzymes, and cholesterol. (**C**) Multi-drug co-loaded nanocarriers for the treatment of atherosclerosis. The red and blue triangles represent different drugs.

Traditional anti-atherosclerotic drugs such as statins and anti-inflammatory agents often cause systemic side effects. Targeted delivery can confine drugs to plaques, reducing dosage requirements. Targeted nanomedicines are drug delivery systems designed using nanotechnology, which employ nanoparticles (typically 20–200 nm in size) as carriers to precisely deliver drugs to atherosclerotic plaques while minimizing damage to normal tissues. The targeting mechanisms include passive targeting via the high permeability of inflamed sites and active targeting via surface-modified ligands that specifically bind to receptors at lesion sites [54]. The premise for designing targeted formulations is to identify specific highly expressed targets in atherosclerotic lesions (Figure 3A). The markers and cells that have been targeted with nanoparticles are summarized in Table 1. Given the complex pathogenesis of AS, validated targets currently mainly include incomplete endothelial cells [55], vascular smooth muscle cells (VSMCs) [56], macrophages [57], and the extracellular matrix at lesion sites [58]. Additionally, neutrophils continuously accumulating in AS sites are also target cells [59].

##### Ligand-Modified Nanoparticles Designed for Specific Lesion Sites

Endothelial cells in the AS site are different from normal tissues and are the targets of nanomedicines. Clinical observations showed that AS preferentially develops in curved and branching arterial regions, where endothelial cells are exposed to disturbed blood flow with characteristic low-magnitude oscillatory shear stress [60]. When endothelial cells are exposed to disturbed blood flow, their biological behavior undergoes significant changes. Endothelial cells no longer align and elongate in a specific direction but instead exhibit a pro-inflammatory and pro-thrombotic phenotype [61]. This phenotypic switch is closely linked to the sustained activation of pro-inflammatory signaling pathways, manifested by significantly increased expression of inflammatory mediators such as vascular cell adhesion molecule-1 (VCAM-1), intercellular adhesion molecule-1 (ICAM-1), and monocyte chemoattractant protein-1 (MCP-1) [62]. This phenomenon provides unique biological targets for designing targeted therapeutic strategies. Among these, VCAM-1 is highly expressed after the activation of vascular endothelial cells, mediates the adhesion and migration of monocytes into the vascular wall, and promotes plaque formation [63]. Zhou et al. [64] previously designed VCAM-1-targeted polyelectrolyte complex micelles loaded with the targeting peptide VHPKQHR. This system demonstrated targeted effects in in vivo AS treatment. Another study showed that nanoparticles binding with VCAM-1 expression at plaque sites can also inhibit the recruitment of inflammatory cells to plaques [65]. They further demonstrated that targeting VCAM-1 can synergistically enhance the anti-inflammatory effects of IL-10 and other therapies. Therefore, leveraging VCAM-1’s specific high expression in atherosclerotic plaques and its critical role in immune cell infiltration has emerged as a promising therapeutic strategy. Furthermore, in vivo phage display methods have enabled the development of targeted peptide molecules for blood flow-disturbed regions, offering new insights for early prevention and treatment of AS. Phage display technology involves fusing foreign polypeptide or protein genes with phage coat protein genes, enabling target molecules to be displayed on the phage surface [66]. Within the complex in vivo physiological environment, this technology screens phage clones that can specifically bind to target tissues, cells, or molecules, thereby obtaining biomolecules with targeting functions [67]. Hofmeister et al. [68] successfully screened four peptide molecules that specifically target the vulnerable vascular system of AS using in vivo phage display. A targeted liposomal nanoparticle constructed with the candidate peptide PREY exhibited active enrichment in shear-stress-disturbed regions and was confirmed to precisely localize to endothelial cells in the vulnerable vascular system of AS, providing a highly promising candidate for targeted therapy of endothelial dysfunction.

VSMCs play a central role in plaque formation and evolution during AS, with their phenotypic transformation established as a promising therapeutic target. Specifically, in the early stages of lesion development, quiescent contractile VSMCs undergo phenotypic conversion to a synthetic phenotype, acquiring excessive proliferative capacity and migrating to the vascular intima [56]. As the disease progresses, VSMCs further transdifferentiate into alternative phenotypes with characteristics of macrophages, foam cells, and osteochondrogenic cells. Notably, VSMCs highly express C-C chemokine receptor 2 (CCR2) under pathological conditions, providing a key theoretical basis for developing VSMC-targeted therapeutic strategies [69]. As a high-affinity ligand for CCR2, MCP-1 (also known as CCL2) specifically recognizes and binds to CCR2 highly expressed on the surface of synthetic VSMCs [70]. Based on this mechanism, Chin et al. [71] designed a novel delivery system for targeting AS smooth muscle cells. The nanoparticles modified with the MCP-1 peptide were loaded with miR-145 to form micelles, which specifically bind to CCR2 for targeted drug delivery, enabling precise delivery of therapeutic miRNAs to atherosclerotic lesion sites.

Foam macrophages are the hallmark cells of AS. During AS progression, monocytes migrate into the vascular intima and differentiate into macrophages, which form foam cells after phagocytosing oxidized low-density lipoprotein (oxLDL), constituting the main component of the plaque lipid core [72]. Foam cells cannot migrate effectively, accumulating within plaques and releasing pro-inflammatory factors, which exacerbate local inflammation and promote VSMCs phenotypic transformation and plaque progression [73]. As iconic cells in atherosclerotic sites, foam macrophages are rich in oxLDL and highly express scavenger receptors (e.g., SR-A, CD36), making them ideal targets for nanoparticles [74]. Natural apoA-I, a major component of HDL responsible for cholesterol efflux from foam cells, inspired researchers to design the 4F peptide (sequence: FAEKFKEAVKDYFAKFWD) based on its structure [75]. Retaining the α-helical structure of apoA-I, the 4F peptide mimics the lipid-binding and reverse cholesterol transport functions of HDL, targeting oxLDL-rich foam macrophages and directly intervening in their cholesterol metabolism. Additionally, foam cells in atherosclerotic plaques highly express the p32 receptor due to oxLDL uptake. The cyclic peptide Lyp-1 (CGNKRTRGC), which binds to the p32 receptor, has been identified as a valuable tool with remarkable ability to penetrate atherosclerotic plaques and target foam macrophages. Studies showed that Lyp-1 modified liposomes exhibit 25-fold higher fluorescence signal intensity in mouse aortic plaques compared to non-targeted groups.

Macrophages are immune cells in AS development, with their functional states directly influencing plaque stability and disease progression. Macrophages are primarily categorized into pro-inflammatory (M1) and anti-inflammatory (M2) types. Early plaques are dominated by M2 macrophages, promoting fibrous cap thickening; advanced plaques have higher M1 macrophage prevalence, leading to uncontrolled inflammation and plaque vulnerability [76]. S2P specifically binds to the overexpressed Stabilin-2 receptor on macrophage surfaces, which is significantly upregulated in M1 macrophages of atherosclerotic plaques but minimally expressed in normal tissues, enabling lesion-specific accumulation. The macrophage-targeting peptide ligand S2P (Cys-Arg-Leu-Thr-Leu-Thr-Val-Arg-Lys-Cys, CRTLTVRKC), which specifically binds to the Stabilin-2 receptor on pathological macrophages, holds significant value in targeted AS therapy [77]. Huang et al. [78] developed S2P ligand-modified siRNA-loaded nanoparticles, demonstrating that S2P50 nanoparticles exhibit 3-fold higher gene silencing efficiency than non-targeted S2P0 nanoparticles, highlighting the ligand’s role in enhancing delivery efficiency. During apoptosis, phosphatidylserine (PtdSer) is externalized to the cell membrane surface as an “eat me” signal, recognized and phagocytosed by PtdSer receptors (e.g., Tim-4, Bai1) on macrophages. Wu et al. [79] developed apoptotic body-mimetic liposomes (AP-Lipo) by modifying liposome surfaces with PtdSer to selectively deliver pioglitazone (PIO) to atherosclerotic macrophages. PtdSer-mediated “eat-me” signaling enhanced M1 macrophage uptake, with ApoE−/− mouse models showing 4-fold higher fluorescence intensity in plaques for AP-Lipo compared to controls. In addition to modifying nanoparticles with ligands to achieve targeting functionality, directly formulating target-functional ligands into nanoparticles represents a strategy with greater clinical translation value. A recent study revealed that hypoxia, rather than pro-inflammatory cytokines, increases glucose uptake in human macrophages. Majmudar et al. [80] developed 13 nm dextran nanoparticles (DNP) to investigate macrophage targeting, showing that DNP is preferentially internalized by monocytes and macrophages via phagocytosis. Flow cytometry indicated that 76.7% of DNP signals originated from monocytes/macrophages, significantly higher than neutrophils (11.8%) and lymphocytes (0.7%). Ferritin, an iron storage and transport protein, accumulates in macrophages of human atherosclerotic plaques. Uchida et al. [81] utilized ferritin as an intrinsic nanoplatform to target imaging agents to vascular macrophages for detecting high-risk atherosclerotic plaques. Engineered human ferritin cages, conjugated with fluorescent Cy5.5 or encapsulated with magnetite nanoparticles, were internalized by macrophages in mouse atherosclerotic carotid arteries and visualized via fluorescence and magnetic resonance imaging.

The atherosclerotic extracellular matrix (ECM) plays a critical role in plaque formation, progression, and vulnerability, with its dynamic remodeling and compositional changes directly impacting the vascular microenvironment and plaque stability [82]. The atherosclerotic ECM primarily consists of collagen, elastic fibers, and polysaccharides. Proteoglycans like heparan sulfate and hyaluronic acid (HA) promote lipid retention and oxidative stress by electrostatically adsorbing low-density lipoprotein (LDL), accelerating lipid core formation [83,84]. During AS pathogenesis, overexpression of HA receptors (e.g., Stabilin-2, CD44) confers potential on HA nanoparticles (HA-NPs) for active targeting of AS lesions [85]. Collagen, a core ECM component, provides structural support and mechanical strength to blood vessels. In atherosclerotic lesions, collagen fiber structure gradually disorganizes and decreases in content, leading to thinning of the fibrous cap and increased plaque vulnerability [86]. Stable plaques contain significantly more collagen than vulnerable plaques. This protein is excessively exposed at vascular rupture sites, with over 50% of lesion bases composed of collagen IV (Col IV). Through phage display studies, Kamaly et al. [87] identified the peptide sequence KLWVLPK with affinity for Col IV. They used biodegradable polymers (PLA, PLGA, PEG) combined with Col IV-targeting peptides to form core–shell-structured NPs, anchoring IL-10-loaded NPs within mouse atherosclerotic plaques via this targeting strategy. Atherosclerotic plaque rupture exposes collagen and other plaque components to blood, initiating vascular hemostasis, thrombin activation, and thrombus formation at rupture sites, leading to fibrin deposition inside and on the surface of plaques [88]. Deshpande et al. [89] tested the clot-binding peptide cysteine–arginine–glutamic acid–lysine–alanine (CREKA) for targeting fibrin within plaques, demonstrating that CREKA-targeted micelles enable drug delivery in an ApoE−/− mouse model.

##### Cell Membrane-Coated Nanoparticles

Cell membrane-coated nanoparticles (CMNPs) represent an innovative biomimetic nanotechnology that endows synthetic nanoparticles with unique biological functions, such as immune evasion, target recognition, and inflammation tropism, by coating their surfaces with natural cell membranes [90]. The membrane components from specific cells (e.g., macrophages, neutrophils, platelets) are extracted and coated onto polymer or lipid nanoparticles, preserving native membrane proteins and receptor functions. This technology offers significant advantages in AS-targeted therapy.

Macrophages represent large-sized and highly adaptable white blood cells that naturally serve as primary cellular effectors in inflammatory responses and tissue repair mechanisms. These cells possess the capabilities to transverse physiological barriers, evade immune detection, facilitate intracellular transport, and home to inflamed sites [91]. Zhu et al. [92] developed macrophage membrane-coated nanoparticles MM@DA-pCD@MTX, where CD47 protein on the MM surface inhibits macrophage phagocytosis via “don’t eat me” signaling, extending blood circulation half-life from 1.87 to 4.38 h. In another study, researchers demonstrated that macrophage membrane-coated nanoparticles MM/RAPNPs retained integrin α4β1 on their surface for VCAM-1 targeting, showing 2-fold higher fluorescence signal intensity in aortic plaques of ApoE−/− mice compared to RAPNPs [93].

Neutrophils, as natural “inflammation sentinels,” actively migrate to inflamed sites, and their membrane-coated nanoparticles inherit this property. Neutrophil membranes carry “self” surface molecules (e.g., CD47, CD11b/CD18) that bind to inhibitory receptors (e.g., SIRPα) in the immune system, transmitting “non-foreign” signals to suppress phagocytosis by macrophages and dendritic cells [94]. Additionally, chemokine receptors (e.g., CXCR1, CXCR2) and adhesion molecules (e.g., L-selectin, αLβ2 integrin) on neutrophil membranes specifically recognize abundant inflammatory factors (e.g., IL-8, CCL2) and activated vascular endothelial cells (highly expressing ICAM-1, VCAM-1, P-selectin) at atherosclerotic lesions, guiding nanoparticles to migrate and accumulate in inflamed areas [95]. For example, neutrophil membrane-coated nanoparticles (NeuM@P5c/S/C) showed 2-fold increased plaque accumulation due to LFA-1/CD18-mediated inflammation targeting [96].

Platelets, crucial for hemostasis, are closely associated with atherosclerosis at multiple stages and exhibit significant crosstalk with inflamed endothelium [97]. Their hemostatic properties require strong binding to exposed subendothelial matrices. They also bind immune cells and recruit them to inflamed sites [98]. Taking advantage of the tendency characteristics of platelets towards atherosclerotic plaques, researchers coated platelet-derived membranes onto fluorescent dye-loaded poly(lactic-co-glycolic acid) (PLGA) nanoparticles to create PNP [99]. Results showed significant fluorescence enrichment of PNP in aortic plaques of ApoE KO mice, while PEG-modified PLGA nanoparticles exhibited no specific distribution.

##### Neutrophil Hitchhiking Nanoparticles

In situ neutrophil hitchhiking is a promising strategy to leverage neutrophils’ innate chemotaxis for efficient delivery of nanomedicines and diagnostic reagents to inflamed/autoimmune sites [100]. Due to overexpression of integrin ανβ3 on neutrophil surfaces, cRGD-modified nanoparticles can be endocytosed by blood neutrophils, enabling neutrophil hitchhiking. In one study, cRGD was used to modify sivillistat sodium (SVT)-loaded liposomes, constructing a neutrophil hitchhiking system (cRGD-SVT-Lipo) for targeted SVT delivery [101]. cRGD-SVT-Lipo releases SVT in plaques or neutrophils, inhibiting neutrophil elastase intracellularly and extracellularly to combat atherosclerosis. In vivo distribution showed 2.5-fold higher fluorescence intensity of cRGD-SVT-Lipo in ApoE−/− plaques compared to unmodified liposomes. After 3 weeks of treatment, the cRGD-SVT-Lipo group exhibited a 45% reduction in aortic root plaque area, significantly outperforming free SVT.

##### Ligand-Free Targeted Nanoparticles

Traditional strategies often rely on ligand modification (e.g., antibodies and peptides) to enhance targeting but face challenges like immunogenicity and complex preparation. Recent studies showed that targeted delivery can be achieved without additional ligands by regulating nanoparticle composition or morphology, with advantages being deposition in lesions without specific receptor expression [102]. Yi et al. [103] explored enhancing dendritic cell (DC) targeting in AS by adjusting nanostructure morphology (without surface ligand modification), revealing the impact of morphology on immune cell-specific uptake and its therapeutic potential. Three morphologies were self-assembled from PEG-bl-PPS block copolymers, including ~20 nm spherical micelles (MC), ~100 nm polymeric vesicles (PS), and ~50 nm fibrous micelles (FM). Results showed that PS morphology was critical for targeting DCs, particularly in the spleen and AS lesions, offering new strategies for cardiovascular immunotherapy.

**Table 1:** Examples of nanoparticles targeting atherosclerotic plaque.

Target	Targeting Moiety	Nanocarrier	Cargo	Model	Ref.
VCAM-1	VHPKQHR	micelles	miR-92a inhibitors	ApoE−/−	[64]
CCR2 expressed on synthetic VSMCs	MCP-1 peptide	micelles	miR-145	ApoE−/−	[71]
Macrophage receptor stabilin-2	Macrophage-targeting peptide ligand S2P	polymer-lipid hybrid nanoparticles	CaMKIIγsiRNA	Ldlr−/−	[78]
oxLDL-rich foamy macrophages	Phase-changing peptide (FFFFFFFFFFGDWFKAFYDKVAEKFKEAF)	nanoemulsions	Simvastatin	ApoE−/−	[75]
Col IV	Col IV targeted peptide	polyester polymers	IL-10	Ldlr−/−	[87]
Fibrin clots	The peptide CREKA (Cys-Arg-Glu-Lys-Ala)	nanoemulsions	17-β-estradiol	C57BL/6	[89]
Atherosclerotic macrophages	PtdSer	liposome	PIO	ApoE−/−	[79]
Fibronectin and Filamin-A expressed by endothelial cells in the interfered area of blood flow	The peptide GSPREYTSYMPH (PREY)	liposome	tetrahydrobiopterin	ApoE−/−	[68]
The p32 receptor expressed on foam cells	Cyclic peptide Lyp-1 (CGNKRTRGC)	liposome	GW3965	Ldlr−/−	[104]
The IL-4 receptor	Atherosclerotic plaque-homing peptide (AP peptide)	hydrophobically modified glycol chitosan	Cy5.5	Ldlr−/−	[105]
Stabilin-2 and CD44	HA	HA nanoparticles	Cy5.5	ApoE−/−	[85]

Abbreviations: VCAM-1, vascular cell adhesion molecule 1; CCR2, C-C chemokine receptor-2; Col IV, collagen IV; MCP-1, monocyte chemoattractant protein-1; PtdSer, phosphatidylserine; HA, Hyaluronic acid; IL-10, interleukin-10; PIO, pioglitazone.

#### 3.2.2. Stimulus-Responsive Nanoparticles

In recent years, microenvironment-responsive nanodrug delivery systems have attracted increasing attention from researchers. These stimulus-responsive drug delivery systems are designed to trigger the release of contents from nanoparticles in an “intelligent” manner by leveraging changes in physiological conditions at lesion sites under disease states [106]. This overcomes the drawback of premature drug release in traditional nanoparticles, improves drug bioavailability, prolongs blood circulation time, and enhances overall therapeutic efficacy. With the deepening of research on inflammatory mechanisms, the unique pathophysiological characteristics of AS lesion sites, such as blood flow shear stress [60], hypercholesterolemia [107], microacidity [108], reactive oxygen species (ROS) [109], and abnormal enzyme elevation [110], have received extensive attention (Figure 3B).

##### ROS

ROS plays a non-negligible role in the development of AS. ROS are generated during mitochondrial oxidative metabolism, which are crucial for maintaining vascular homeostasis. However, excessive production of ROS can cause a series of events closely related to AS development, such as endothelial damage, LDL oxidation, foam cell formation, monocyte migration, and degradation of the fibrous wall [109,111]. Therefore, developing ROS-responsive nanodrug delivery systems targeting AS is an effective therapeutic strategy, which can not only scavenge excessive ROS in the AS disease microenvironment but also achieve controlled drug release.

Many ROS-responsive chemical groups can be used for designing stimulus-responsive nanomaterials, including hydroxymethylferrocene (Fc) [112], thioacetal linkers (TKL) [113], thioether groups (-S-) [114], oxalate esters [115], and phenylboronic esters [116]. Polymers containing these ROS-sensitive linkages can self-assemble into nanoparticles, and drugs can be loaded inside the nanoparticles or bonded to the surface of nanoparticles via ROS-sensitive chemical bonds. Among these ROS-sensitive linkages, oxalate esters have been widely studied to design ROS-sensitive nanoparticles for AS treatment. Oxalate ester bonds covalently couple the hydroxyl groups of polymers and drugs using oxalyl chloride. Under the action of ROS, the oxalate ester bond can degrade into non-toxic CO_2_ and release the parent drug. For example, Yang et al. [117] designed ROS-responsive simvastatin (SV) nanopro-drugs (OPDH-SV) by conjugating SV and tertiary amine oxide zwitterionic polymers (OPDH) through oxalate ester bond. Utilizing their ROS-responsive drug release properties, they released drugs at AS lesions and reduced the hepatotoxicity of SV. However, oxalyl chloride, required for synthesizing oxalate esters, is highly toxic, which may pose challenges in large-scale production. Phenylboronic esters are another type of ROS-responsive linkage. The boron atom in phenylboronic esters acts as a Lewis acid, binding to the hydroxyl oxygen in adjacent or meta-diol structures to form five- or six-membered cyclic borates. When encountering ROS (such as H_2_O_2_ and ·OH), ROS insert into the C-B bond of the borate, causing the bond to break, generating phenol and borate derivatives, and releasing the loaded drug or functional molecules simultaneously [118]. Using this property, Li et al. [119] combined hyaluronic acid with phenylboronic esters (H_2_O_2_-responsive) to deliver methotrexate (MTX), achieving lesion-specific drug release. In addition, TKL linkages have also attracted the attention of researchers. The structure of TKLs is formed by connecting two sulfur atoms through an acetal bond (R-S-C-R′-S-R″). In the microenvironment of inflammation, excessive ROS attack the C-S bond of TKL, leading to linker cleavage, generating ketones and thiol derivatives, and releasing loaded drugs or functional molecules. Using this property, researchers constructed liposomes with DSPE-TKL-PEG2000 to deliver geniposide (GP) and emodin (EM). The TKL bonds break in high-ROS environments, triggering drug release [120].

In addition to using ROS-sensitive linkages to break and release drugs under the action of ROS, another strategy for constructing drug delivery systems using ROS-responsive properties is to generate hydrophilic groups through the reaction of ROS-sensitive chemical bonds with ROS, thereby changing the hydrophilic-lipophilic balance of polymers and promoting the dissociation and drug release of the drug-loaded system. For example, thioether groups (-S-) in polypropylene sulfide are introduced into the polymer through ring-opening polymerization, and their structure is ROS-responsive. Thioethers can be oxidized by hydrogen peroxide (H_2_O_2_) to hydrophilic sulfoxides (-SO-) or sulfones (-SO_2_-), converting the originally hydrophobic thioether segments into hydrophilic structures [114], thereby disrupting the hydrophobic core of the micelles and triggering micelle disassembly. Based on this property, Shen et al. [121] designed a glycidyl methacrylate-polysulfide micelle to deliver simvastatin, which is a water-insoluble drug. These micelles undergo oxidation in the high-ROS environment of AS lesions, converting thioether groups into hydrophilic groups, leading to the rupture of micelle structures and the release of simvastatin. Similarly, another typical system is based on the ROS-responsive properties of hydroxymethylferrocene (Fc). The molecular structure of Fc is a planar sandwich structure with an iron atom sandwiched between two cyclopentadienyl groups. The Fe^2+^ in the ferrocene core can catalyze the decomposition of H_2_O_2_ through the Fenton reaction, generating highly active hydroxyl radicals (·OH) and converting to Fe^3+^. This process is accompanied by a significant change in molecular polarity, enhancing the hydrophilicity of nanoparticles. Hou et al. [112] utilized this property to construct nanoparticles containing Fc structures for delivering hydrophobic curcumin. At AS lesion sites, Fc undergoes a polarity shift in response to locally high concentrations of ROS, enhancing the hydrophilicity of nanoparticles and thus promoting the release of hydrophobic curcumin.

##### pH

Naghavi et al. measured the pH range of atherosclerotic plaques in humans and rabbits using two pH-sensitive fluorescent dyes, finding values of approximately 6.5–8.5 and 5.5–7.5, respectively [108]. It is now widely accepted that the atherosclerotic microenvironment is weakly acidic. The accumulation of highly oxygen-consuming foam cells in atherosclerotic lesions, particularly in advanced plaques, leads to insufficient oxygen supply from luminal blood and vasa vasorum. Increased oxygen demand and inadequate supply result in severe hypoxia in macrophage-rich regions. Macrophages adapt to hypoxia by efficiently generating ATP through anaerobic glycolysis, but extensive glycolysis also promotes lactic acid accumulation (lactic acid concentration > 10 mM) within lesions, lowering the pH of the pathological environment [122]. The microenvironment of atherosclerotic plaques is reported to be weakly acidic, with a pH range of 6.0–6.8 [123]. Another study showed that extracellular microenvironmental acidification (pH 5.5) occurs in advanced atherosclerotic plaques [124].

Leveraging the low-pH characteristics of AS lesion sites, pH-sensitive release nanoparticles can be designed for precise drug delivery. Hydrazone bonds, important pH-sensitive chemical linkages, are widely used in designing intelligent pH-responsive drug delivery systems. Formed by the condensation of carbonyl (C=O) and hydrazine (-NH-NH_2_) groups, hydrazone bonds undergo cleavage in acidic environments (pH ≤ 5.5) as H^+^ attacks the carbon-nitrogen double bond, generating ketones and hydrazine groups [125]. Using this property, Cheraga et al. [126] covalently linked all-trans retinoic aldehyde (ATR) to hyaluronic acid (HA) via hydrazone bonds to form an amphiphilic polymer, with HA as the hydrophilic shell and ATR as the hydrophobic core. Under weakly acidic plaque microenvironment conditions (pH 6.5), drug release was slow, with 33.5% ATR released. After cellular uptake, more complete drug release occurred in the pH 5.2 lysosomal environment, 80.3% ATR were released, achieving efficient intracellular delivery. Similarly, benzoic-imine (BI) linkers [127], formed by the condensation of benzaldehyde and amino compounds, undergo rapid hydrolysis in acidic environments (pH 5.6) through the dynamic reversibility of imine (C=N) bonds, generating benzoic acid and amines that are easily metabolized, reducing toxicity risks. Lin et al. [128] constructed nanoparticles with BI linkers for simvastatin (ST) delivery. Due to pH-triggered local release, the intralesional concentration of ST was 1000-fold higher than that of free drug. In addition to covalent chemical bonds for acid-responsive release, non-covalent coordination bonds have been used to design acid-responsive nanoparticles [129]. For instance, a drug delivery system is constructed through non-covalent conjugation of tannic acid (TA) and Mn^2+^ to form a metal-phenolic network (MPN) structure [130]. In the weakly acidic microenvironment of atherosclerotic lesions, H^+^ competitively binds to the phenolic hydroxyl groups of TA with Mn^2+^, disrupting coordination bonds and resulting in MPN disintegration with subsequent release of AS therapeutic agents.

##### Blood Flow Shear Stress

Hemodynamic factors such as blood flow shear stress (FSS) have increasingly garnered attention from scholars for their impact on the stability of atherosclerotic plaques. It is currently the consensus that the most frequent site for carotid atherosclerotic plaques is the carotid artery bifurcation, with nearly 95% of lesions located within 2 cm of the bifurcation origin [131]. Blood flow in the common carotid artery is unidirectional (from the heart to the brain) and laminar. Due to the bifurcation of the vessel diameter, the blood flow direction suddenly changes, diverging into the internal and external carotid arteries, creating high and low shear stress regions at the bifurcation. This leads to unstable hemodynamic conditions, making lipids prone to deposition and causing damage to the vascular intima due to blood flow impact. Consequently, platelet and collagen aggregation are triggered, promoting the formation of atherosclerotic plaques and mural thrombi [132]. In the carotid bifurcation, hemodynamic conditions vary due to uneven plaque thickness. Taking advantage of this characteristic of the lesion site of AS, researchers designed the nanocarriers displaying sensitivity to shear stress, such as liposomes [133], micelles [121], and aggregates [134]. Holme et al. [135] have designed a shear stress-responsive nanoparticle based on the hemodynamic characteristics of AS. Pad-PC-Pad is a synthetic phospholipid with a phosphocholine polar head group, in which the 1,3 positions of the glycerol backbone are substituted with palmamide to form a symmetric 1,3-diamide structure. Results show that Pad-PC-Pad vesicles (LUVET100) exhibit a biconcave lens shape, with a flat central region and higher curvature at the edges. The equatorial region of the lens-shaped vesicles has the highest curvature and maximum membrane tension. Under shear stress, this region is prone to local deformation, forming transient pores and leading to drug release. After the shear stress ceases, the membrane self-repairs to restore integrity.

##### Enzyme

A notable physicochemical feature in the late stage of AS development is the high expression of multiple proteases at plaque sites, leading to extracellular matrix (ECM) remodeling and fibrous cap degradation, which reduces plaque stability. Enzymes such as matrix metalloproteinases (MMPs) [136], hyaluronidase (HAase) [137], and cathepsins [110] collectively contribute to the initiation and progression of AS. These enzymes are significantly more highly expressed in atherosclerotic lesions than in normal vascular environments. In recent years, using enzymes as endogenous responsive targets for drug delivery has become a research hotspot. For example, Zhang et al. [138] designed a kind of hyaluronic acid-coated nanoparticles, which were degraded and released drugs in the presence of HAase.

Cathepsin K (CTSK), a member of the papain-like cysteine protease family, is synthesized intracellularly as a precursor, activated under acidic lysosomal conditions, and secreted extracellularly, where it degrades the extracellular matrix. Studies have shown that CTSK is highly expressed in AS plaques and is closely associated with AS development [139]. CTSK has the ability to specifically recognize and degrade type I collagen, thereby participating in the remodeling of fibrous plaques by degrading the extracellular matrix and accelerating plaque rupture [140]. Due to these characteristics, CTSK can also serve as a target and biomarker for lesion sites in AS treatment. Therefore, CTSK is a promising candidate for triggering drug release from nanocarriers in atherosclerotic lesions. Based on the finding that CTSK is enriched in atherosclerotic lesions, Fang et al. [141] designed and synthesized a type I collagen-derived peptide sequence (HPGGPQ) as a CTSK-responsive sequence. This sequence was further engineered into a CTSK-responsive nanodelivery system, which effectively inhibits AS progression through local controlled drug release.

##### Cholesterol

Cholesterol crystals are a critical component of atherosclerotic plaques, with their formation closely linked to abnormal metabolism of low-density lipoprotein cholesterol (LDL-C) [142]. When LDL-C penetrates beneath the damaged arterial intima, it becomes oxidized and gradually deposits. As cholesterol transitions from a liquid to a solid crystalline state, its volume can expand by over 45% [143]. This expansion force directly tears the fibrous cap or intimal surface of the plaque, leading to plaque rupture and serving as a key factor in plaque instability. The hypercholesterolemic characteristics of atherosclerotic lesions can be utilized to design a cholesterol-responsive drug delivery system. Cyclodextrins (CDs) are cyclic oligosaccharides formed by glucose units linked via α-1,4-glycosidic bonds, exhibiting a conical cylindrical shape. Their internal hydrophobic cavity is constituted by C-H bonds of glucose units, while external hydroxyl groups render the surface hydrophilic. This “hydrophilic exterior, hydrophobic interior” property allows CDs to encapsulate guest molecules through hydrophobic interactions and van der Waals forces, making them particularly suitable for binding cholesterol [144]. Kim et al. [145] have developed cholesterol-sensitive nanoparticles (CSNPs) based on the “cargo exchange” strategy using CDs as a drug carrier. They used CDs to load statin drugs to create CSNPs. When interacting with cholesterol at lesion sites, CSNPs have a higher affinity for cyclodextrin than statins. Therefore, CSNPs release statins and clear cholesterol through cargo exchange, effectively inhibiting the progression of AS.

#### 3.2.3. Multifunctional Synergistic Nanoparticles

One advantage of using polymer nanomaterials as drug delivery carriers is the ability to achieve combination therapy by simultaneously carrying multiple drugs [146]. This advantage enables the integration of imaging agents and drugs for theranostics, as well as the loading of multiple drugs targeting different sites to achieve synergistic therapeutic effects (Figure 3C).

##### Theranostic Nanoparticles

Reliable diagnostic strategies are critical for improving AS management. Intravascular ultrasound (IVUS) and computed tomography angiography (CTA) are clinical gold standards for visualizing atherosclerotic plaques [147,148]. However, invasive IVUS is prone to causing damage and complications [149]. Due to the rapid flow of iodinated contrast agents in arteries, CTA can only assess arteries larger than 1 mm in diameter, making microvessels commonly present in plaques undetectable [150]. Developing new theranostic nanocarriers holds significant value for AS treatment.

Magnetic Resonance Imaging (MRI) has become one of the gold standards for AS diagnosis due to its non-invasiveness, high resolution, and functional analysis capabilities. MRI generates high-resolution images using magnetic fields and radiofrequency pulses without radiation exposure. The non-invasive, painless procedure with strong reproducibility provides safe support for long-term monitoring of plaque progression [151]. MRI can display arterial wall thickness, plaque morphology, and location with submillimeter resolution. Gadolinium (Gd^3+^), iron, and manganese are core elements for MRI enhancement, optimizing signal contrast through paramagnetic or superparamagnetic mechanisms [152]. Lin et al. [128] designed nanoparticles with porous structures using benzoic-imine (BI) linkers and Gd^3+^, with simvastatin (ST) adsorbed within the internal pores and outer lipid bilayers of the nanoparticles via hydrophobic interactions (ST/NCP-PEG). After intravenous injection, ST/NCP-PEG nanoparticles significantly accumulated in aortic plaques of ApoE−/− mice, with peak Gd^3+^ content in plaques, and MRI clearly visualized plaque locations.

Two-Photon Excitation Aggregation-Induced Emission (TPE-AIE) is a novel optical material technology combining two-photon excitation (TPE) and aggregation-induced emission (AIE) properties. Its core mechanism involves exciting materials via simultaneously absorbing two low-energy photons and leveraging the enhanced luminescence of AIE materials in aggregated states to address challenges in biological imaging, such as penetration depth, photostability, and background interference [153]. TPE refers to the process where molecules absorb two long-wavelength (typically near-infrared) photons simultaneously to transition to an excited state. Compared to single-photon excitation, TPE uses longer wavelengths, significantly improving tissue penetration depth while reducing photodamage and background fluorescence interference [154]. AIE materials exhibit weak fluorescence in single-molecule states but enhanced luminescence in aggregated states due to restricted intramolecular motion, avoiding the aggregation-caused quenching (ACQ) problem of traditional fluorescent materials [155]. For example, a two-photon AIE fluorophore was conjugated to β-cyclodextrin (CD) via thioether bonds, with the CD cavity encapsulating the anti-inflammatory drug prednisolone (Pred) through host-guest interactions to form the two-photon fluorophore-cyclodextrin/Pred complex (TPCDP) [156]. After administration, significant fluorescence signals were observed in aortic plaques of ApoE−/− mice, with two-photon imaging clearly visualizing three-dimensional plaque structures.

Ultrasound technology has become an important tool for AS diagnosis and assessment due to its non-invasiveness, real-time capability, and cost-effectiveness. Despite being a primary diagnostic technique with proven utility in AS detection, early-stage plaque detection remains challenging [157]. The emergence of microbubble contrast agents has improved ultrasound imaging sensitivity and resolution, making it an ideal technology for disease diagnosis [158]. Additionally, microbubbles can serve as effective drug delivery carriers. However, their large particle size significantly limits therapeutic efficacy, necessitating the development of smaller-scale bubbles such as nanobubbles (NBs). Furthermore, continuous high shear stress in arteries hinders traditional NBs from adhering to blood vessels in target areas. Thus, there is an urgent need to develop actively targeted therapeutic NBs for effective ultrasound imaging and anti-atherosclerotic therapy in early disease stages [159]. In order to solve above challenge, Lin et al. [160] used VCAM-1 modified PLGA nanoparticles to encapsulate a gas core (ammonium bicarbonate), Fe_3_O_4_, and the anti-inflammatory drug R406 (Mag-Tar-DL NBs). After tail vein injection, Mag-Tar-DL NBs accumulated in low shear stress regions under magnetic field and antibody guidance, with contrast signals lasting over 3 min, enabling ultrasound imaging-guided AS treatment.

Photoacoustic Imaging (PAI) is a non-invasive imaging technology combining optical excitation and acoustic detection, based on the photoacoustic effect where light energy is converted to heat, inducing thermoelastic expansion and generating acoustic signals [161]. PAI integrates the high contrast of optical imaging with the deep penetration of ultrasound, enabling non-invasive and dynamic analysis of AS plaque composition, function, and metabolic status. It has demonstrated significant value in precise cardiovascular disease diagnosis and treatment, offering new options for routine early detection of AS plaques in clinical practice. In recent years, various photoacoustic contrast agents (e.g., small molecules, gold nanoparticles, quantum dots, polymers) have been investigated for non-invasive PAI [162]. Among them, π-conjugated polymer probes have been applied to deep tissue imaging due to their excellent PA performance, enabling specific disease diagnosis. In one study, a π-conjugated polymer PMeTPP-MBT was designed as a novel PA contrast agent [163]. On this basis, a smart responsive theranostic nanoplatform (PA/ASePSD) was developed by integrating astaxanthin and SS-31 peptide with loaded PMeTPP-MBT. In an ApoE−/− mouse model of AS, PA signal intensity in plaques reached 1.8 a.u. after injecting PA/ASePSD, which is 6.5 times higher than in normal vessels.

##### Multi-Target Therapy Nanoparticles

The development of AS involves an intertwined pathological network of multiple links, including endothelial injury, lipid metabolism disorders, chronic inflammation, oxidative stress, and immune dysfunction. Single-target therapies, such as statins for lipid regulation, are effective but cannot comprehensively block other key pathological pathways [164]. In contrast, multitarget combination therapy can achieve better efficacy at lower doses and reduce side effects through synergistic actions of different mechanisms. Clinical trials on combination therapies for AS have studied dual-anti-inflammatory, dual-antioxidant, dual-lipid-lowering, and dual-antiplatelet drug combinations, as well as antioxidant + anti-inflammatory and antioxidant + lipid-lowering combinations. Nanoparticles can achieve co-delivery of drugs and enhance therapeutic effects by enabling precise control of drug ratios, release timing, and targeting.

Elevated plasma cholesterol, particularly low-density lipoprotein cholesterol (LDL-C), is a core driver of AS development. While chemical drugs like statins inhibit cholesterol synthesis, they suffer from insufficient lesion accumulation. Although nanotechnology can address these issues, the complex pathogenesis of AS has increasingly highlighted the limitations of single therapeutic strategies in tackling its multifaceted mechanisms, necessitating further enhancement of the therapeutic effects of single drug-loaded formulations [165]. Gene-drug co-loaded nanoparticles exhibit unique advantages in AS treatment by integrating the precise regulatory capabilities of gene therapy with the direct action of chemical drugs. ATP-binding cassette transporters A1 (ABCA1) and G1 (ABCG1) are core molecules that mediate the efflux of cholesterol from macrophages to high-density lipoprotein (HDL). miR-33 can directly bind to the 3’UTR of ABCA1 and ABCG1 mRNA, inhibit their expression, lead to cholesterol accumulation in macrophages, and promote the formation of foam cells. Jiang et al. [166] conjugated atorvastatin to trimethyl chitosan through chemical modification to form an amphiphilic polymer nanoparticles (GTANPs), and anti-miR-33 pDNA was encapsulated into GTANPs via electrostatic interaction, enabling effective co-delivery of statins and nucleic acids. Atorvastatin suppresses endogenous cholesterol synthesis, while pAnti-miR-33 upregulates macrophage ABCA1 expression to promote cholesterol efflux and induce M2 polarization. In an ApoE-KO mouse model, intravenous injection of GTANPs/pAnti-miR-33 reduced aortic plaque area and improved plaque stability. Similarly, He et al. [167] used PAMAM as the nanocore, covalently conjugated LXR ligands (LXR-L) to PAMAM via PEG linkers, and adsorbed siRNA onto the positively charged nanoparticle surface through charge interactions to achieve gene-drug co-loading. SR-A siRNA reduces oxLDL uptake by knocking down scavenger receptor SR-A expression in macrophages, while LXR-L upregulates cholesterol transporters ABCA1 and ABCG1 to promote cholesterol efflux to HDL. In an LDLR−/− mouse model, the total aortic plaque area in the treatment group was significantly reduced. In general, combined inhibition of cholesterol influx with promotion of efflux through multidrug-loaded nanoparticles, providing a new strategy for atherosclerosis regression, especially for advanced plaques unreversed by existing lipid-lowering therapies.

Persistent inflammation is a key determinant of plaque development. Macrophages, the most abundant white blood cells in plaques, play a major role in inflammation by producing pro-inflammatory cytokines [168]. While chemical drugs like aspirin and oridonin can significantly inhibit inflammation, single anti-inflammatory therapy still fails to reduce mortality in AS patients [169]. To enhance the therapeutic effect of anti-inflammatory therapy in AS, an increasing number of studies have designed multidrug-loaded nanoparticles for synergistic enhancement of anti-inflammatory therapy. The targets selected for enhancing the effect of anti-inflammatory treatment mainly include immune regulation, antioxidation and plaque protection, etc. For example, Zhou et al. [170] designed a core–shell nanodelivery system for co-delivery of acetylsalicylic acid and gene drugs to achieve anti-inflammation and immune regulation. Gold-polyethyleneimine (Au-PEI) nanoparticles loaded with short hairpin RNA targeting Siglec-1 (shSiglec-1) via electrostatic adsorption as the inner core, with the shell adsorbing PEI-aspirin (PEI-ASA) copolymers. Aspirin regulates inflammation and lipid metabolism, while shSiglec-1 reduces CD8^+^ T/NKT cells infiltration. In an HFD-fed Apoe−/− mouse model, ASPA NPs block macrophage-T cell interactions by silencing Siglec-1, synergizing with ASA to promote lipid metabolism and anti-inflammation, effectively converting “hot” plaques to a “cold” state and significantly inhibiting plaque progression. This study enhances the therapeutic effect against AS by combining anti-inflammation with immune regulation. Recently, anti-inflammatory and anti-oxidative therapies have attracted special attention due to their crucial role in atherogenesis. Cheraga et al. [126] developed pH-sensitive hyaluronic acid (HA) nanoparticles (HRRAP NPs) for co-delivery of all-trans retinoic aldehyde (ATR) and rapamycin (RAP). ATR exhibits antioxidant activity, while RAP exerts anti-inflammatory effects and inhibits cell proliferation. After HRRAP NPs treatment, the aortic plaque area in mice was reduced to 10.2%, significantly better than free RAP. This study significantly inhibited plaque progression and enhanced stability through the synergistic effects of anti-oxidation, anti-inflammation, and cell proliferation inhibition. The degradation of extracellular plaque-matrix, which disrupts the plaque microenvironment, is another essential factor in promoting plaque advancement. Li et al. [171] reported a pH-sensitive liposome for co-delivery of an anti-inflammatory agent (oridonin, ORD) and a plaque-collagen protectant (marimastat), integrating anti-inflammatory and collagen-protective mechanisms for AS treatment. Marimastat inhibits matrix metalloproteinase (MMP)-mediated collagen degradation, while ORD exhibits anti-inflammatory effects. In HFD-fed Apoe−/− mice treated with pHSLs, aortic sinus plaque area was significantly reduced, plaque collagen content increased, and macrophage polarization toward anti-inflammatory M2 phenotype was promoted. In general, nanoparticles co-deliver multiple drugs, target multiple pathological links of diseases through different mechanisms of action, exert synergistic effects, significantly improve treatment efficiency, and are particularly effective in atherosclerosis, which is complex disease.

### 3.3. Organic Biomimetic Nanoparticles

The green cluster is focused on the organic biomimetic nanoparticles applied for AS treatment. As shown in the green cluster of Figure S1A,B, “inflammation”, “extracellular vesicles”, “exosomes”, and “dysfunctional vascular endothelial cells” are high-frequency terms, and in the temporal distribution map, these represent relatively cutting-edge research areas. Extracellular vesicles (EVs) provide multi-dimensional solutions for AS treatment through advantages such as targeted delivery, immune regulation, and tissue repair. Compared with traditional nanocarriers, EVs exhibit low immunogenicity, are less likely to trigger immune rejection, and offer higher safety. Membrane surface proteins of exosomes, such as integrins and chemokine receptors, endow them with a natural tropism for inflamed and injured sites. EVs can carry drugs, nucleic acids, and proteins to achieve synergistic effects of anti-inflammation, anti-oxidation, and repair [172]. Further classify the relevant literature, the design of EVs as carriers for AS therapy can be mainly categorized into three main types: EVs isolated from specific cells for AS treatment, drug-loaded EVs, and engineered EVs developed through bioengineering approaches.

#### 3.3.1. EVs from Specific Cell Sources

According to the definition of the International Society for Extracellular Vesicles (ISEV), EVs are lipid bilayer-structured particles released by cells that cannot replicate autonomously, primarily including subtypes such as exosomes, microvesicles, and apoptotic bodies [173]. Exosomes are nanoscale vesicles (30–150 nm in diameter) with demonstrable subcellular origins, produced through the cellular “endocytosis-fusion-exocytosis” mechanism [174]. The plasma membrane invaginates to form early endosomes, which encapsulate bioactive substances such as proteins and nucleic acids from the parent cell. Early endosomes mature into multivesicular bodies (MVBs), within which intraluminal vesicles (ILVs) are formed via secondary budding of the endoplasmic membrane. MVBs fuse with the cell membrane, releasing ILVs into the extracellular space via exocytosis to form exosomes [175]. Microvesicles are larger in diameter (100–1000 nm) and are generated through direct budding of the plasma membrane [176]. During cell activation or injury, local lipid reorganization in the plasma membrane and outward protrusion occur to form vesicles [177]. Small GTPases and calcium ion signals activate cytoskeletal proteins to promote vesicle detachment from the plasma membrane [178]. Apoptotic bodies are products of programmed cell death, with a diameter of 50–5000 nm [179].

Based on the purpose of application and the characteristics of samples, various methods for isolating and purifying exosomes have been developed, mainly including ultracentrifugation, density gradient centrifugation, size exclusion chromatography (SEC), polymer precipitation, immunoaffinity, and microfluidic technology. Centrifugation is a classic and commonly used approach, which includes differential centrifugation and density gradient centrifugation [180]. Differential centrifugation sequentially removes cells, cell debris, and large vesicles by gradually increasing the centrifugal force, and finally obtains exosomes through ultra-high-speed centrifugation. Although its operation is relatively simple, this method requires expensive equipment, has a long running time, and the separation efficiency of exosomes is affected by factors such as acceleration, rotor type, and sample viscosity, resulting in low yield and low purity [181]. Density gradient centrifugation, on the basis of differential centrifugation, adds separation media such as sucrose and iodixanol. It realizes the separation of exosomes from impurities such as protein aggregates by allowing vesicles to stay in different gradient layers according to the differences in density and size between vesicles and other biological molecules [182]. This method has high separation purity, and the buffering effect of the medium can also reduce the damage of centrifugal force to exosomes. However, it is more complicated to operate, has a small sample processing capacity, is time-consuming, and is not suitable for large-scale sample separation. Polymer precipitation commonly uses polyethylene glycol (PEG), which competes with exosomes for binding to water molecules, reduces the solubility of the hydration layer on the surface of exosomes, and promotes the precipitation of exosomes under low-speed centrifugation [183]. This method is easy to operate, but PEG can cause coprecipitation of a large number of lipoproteins as well as polymer residues, resulting in low yield and poor purity, which affects subsequent biological activity research. Studies have shown that combining precipitation with size exclusion chromatography (SEC) can achieve higher yield and purity [184]. Size exclusion chromatography (SEC) achieves separation based on the difference in the speed at which substances of different particle sizes pass through porous polymer gel fillers. This method is beneficial for improving plasma protein contamination, and uses gravity separation to avoid exosome aggregation and damage to membrane integrity and biological activity caused by centrifugal force [185]. Its separation efficiency and purity are better than those of centrifugation, making it suitable for multiple studies on exosomes. However, it is time-consuming and cannot distinguish between other exosomes and vesicles of the same size [186]. Immunoaffinity is based on the principle of antigen-antibody immune reaction. For example, the magnetic bead method captures exosomes through the specific binding between antibodies on the surface of magnetic beads and receptors on the surface of exosome membranes [187]. It has a high separation efficiency for exosomes from specific cell sources and is suitable for exosome marker detection and clinical diagnostic research. However, the reaction reagents are expensive, and it is difficult to elute exosomes from magnetic beads. Microfluidic technology is an emerging method that covers a variety of separation technologies based on the physical and chemical characteristics of exosomes. It has the advantage of automation and has potential in biological function research and clinical diagnosis of diseases. However, the cost of equipment and chips is high, the technical standardization is insufficient, and it is still in the laboratory development stage.

**Figure 4:**
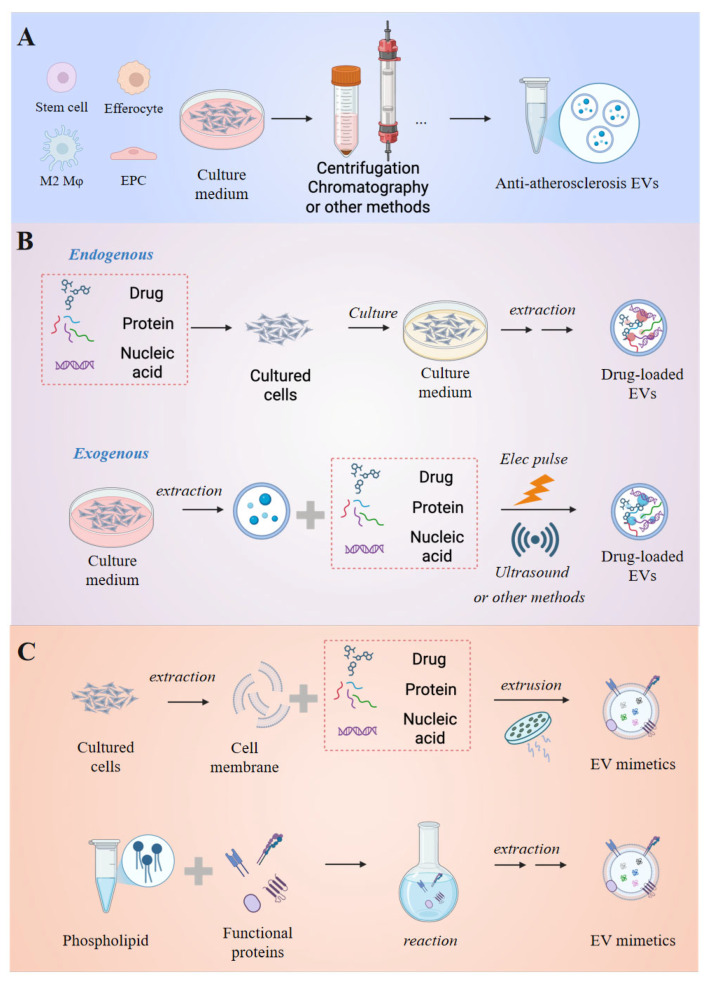
Organic bionic nanocarriers are used for the treatment of atherosclerosis. (**A**) EVs extracted from specific cells (such as stem cells, efferocytes, M2 macrophages, and endothelial progenitor cells) have an anti-atherosclerotic effect. (**B**) Drug-loaded EVs for atherosclerosis therapy, including endogenous and exogenous drug-loading methods. (**C**) The preparation method of EV mimetics.

EVs’ surfaces contain various bioactive molecules, such as extracellular matrix proteins and cell surface receptors, while their contents include nucleic acids, proteins, and other substances, which exhibit specificity depending on the cell of origin [188]. This specificity determines their diverse functions in the treatment of AS. Extensive research has been conducted on the positive and negative effects of EVs from different cell sources on AS development. Some exosomes and microvesicles exert therapeutic effects for AS by regulating mechanisms such as inflammation, oxidative stress, and intercellular communication, while others promote the formation and progression of AS [189,190,191,192,193,194]. This review focuses on EVs with anti-AS effects. As shown in Table 2, we summarize the anti-atherosclerotic effects of EVs from different cell sources. Currently, the cells producing EVs with anti-atherosclerotic properties mainly include: bone marrow mesenchymal stem cells (BMSCs) [195], human umbilical cord mesenchymal stem cells (hUC-MSCs) [196], adipose-derived mesenchymal stem cells (AD-MSCs) [197], adipose-derived stem cells (ADSCs) [198], pluripotent stem cell-derived cells [199], M2 macrophages [200], endothelial progenitor cells (EPCs) [201], and efferocytes [202]. These EVs from different sources exert anti-atherosclerotic therapeutic effects through distinct target mechanisms (Figure 4A).

##### Mesenchymal Stem Cells

Mesenchymal stem cells (MSCs), as pluripotent stromal cells with multi-lineage differentiation potential and immunosuppressive properties, have been recognized as an important source of cells in regenerative medicine [203]. They can be derived from various sources, including bone marrow, adipose tissue, synovium, skeletal muscle, dermis, pericytes, trabeculae, human umbilical cord, lungs, dental pulp, amniotic fluid, fetal liver, and even peripheral blood [204]. The U.S. FDA-approved Remestemcel-L (umbilical cord MSCs) for hormone-resistant acute graft-versus-host disease (GVHD) demonstrates the critical clinical value of these stem cells [205]. Extracellular vesicle therapy based on MSCs has been proven to be biosafe, highly stable and have low immunogenicity. EVs secreted by MSCs have been widely studied for the treatment of AS. The EVs secreted by MSCs can enhance the function of vascular endothelial cells, repair dysfunctional endothelial cells and inhibit plaque formation. For example, in order to solve the issue that endothelial cells (ECs) senescence contributes to AS, Xiao et al. [206] investigated the effects and molecular mechanisms of MSC-derived small EVs (MSC-sEVs) on oxidative stress-induced ECs senescence. They isolated sEVs from MSC culture supernatants via ultracentrifugation and found that these MSC-sEVs highly expressed miR-146a. They demonstrated that MSC-sEVs alleviate oxidative stress-induced EC senescence by regulating the miR-146a/Src pathway. Although this study is significant, it did not investigate the in vivo anti-atherosclerosis effect of MSC-sEVs. Another study explored the mechanism by which MSC-derived exosomes regulate macrophage polarization and migration to clarify their role in AS treatment [207]. After administration in ApoE−/− mouse models, it was found that MSC-derived exosomes overexpressing miR-21a-5p induced macrophage polarization toward the M2 phenotype and significantly reduced aortic plaque area in AS mice. In general, MSC is widely used in tissue repair and for acute and chronic injuries. MSC-derived exosomes may represent a promising therapeutic approach for AS by alleviating endothelial cell aging and reducing inflammation. However, MSC-derived EVs also have problems such as low yield and therapeutic limitations. It may be necessary to screen the most suitable source of MSC for the production of EVs and further verify the long-term safety and therapeutic effect of EV derived from MSC. Problems such as low yield and stability need to be overcome through nanotechnology.

##### Efferocytes

There are a large number of uncleared apoptotic cells in the plaques of advanced AS. When apoptotic cells are not promptly cleared, they exacerbate the inflammatory response and acute event. Efferocytosis is considered the key to eliminating apoptotic cells under physiological and pathological conditions in vivo, which is crucial for maintaining homeostasis. Efferocytes are recruited by chemokines and “come find me” signals and eliminate apoptotic cells through phagocytosis [208]. These efferocytes include bone marrow- and spleen-derived monocytes, differentiated and polarized macrophages, and CD11c DC-like cells. Efferocytes have a unique metabolic pattern that can redistribute excessive lipids and cholesterol after phagocytosis of apoptotic cells, which is of great significance for the treatment of AS. However, the lesion site of advanced AS cannot effectively recruit efferocytes [209]. Compared with the clearance of ACs in other tissues, the clearance function of macrophages to ACs in diseased vessels was significantly impaired, and the phagocytic ability decreased by nearly 20 times. Research showed that efferocyte-derived EVs can increase macrophage efferocytosis efficiency. Bhattacharya et al. [202] reported that efferocyte-derived EVs express prosaposin, which binds to GPR37 on immature macrophages. Through an ERK-AP1-dependent signaling axis, this interaction increases the expression of the endocytic receptor Tim4, enhancing macrophage phagocytic efficiency and accelerating inflammation resolution. Administration of efferocyte-released EVs in ApoE−/− mouse models promotes inflammation regression and tissue repair. Therefore, activating the clearance of apoptotic cells at the lesion site of AS through EVs secreted by efferocytes is expected to become a new approach for the treatment of AS.

##### M2 Macrophages

M2-type macrophages are a functional phenotype formed by the polarization of macrophages in a specific microenvironment. The main functions of M2 macrophages are to suppress inflammation, clear cell debris and apoptotic cells, and promote tissue repair. In the early stages of atherosclerosis or in stable plaques, M2-type macrophages are predominantly distributed in the fibrous cap and adventitial regions, exerting repair functions [210]. The efferocytosis capacity of M2 macrophages is significantly stronger than that of M1 macrophages. By recognizing the “eat-me” signals (such as phosphatidylserine) on the surface of apoptotic cells, M2 macrophages can efficiently clear apoptotic foam cells, reduce the expansion of the necrotic core, and maintain plaque stability. In addition, M2-type macrophages can secrete anti-inflammatory factors such as IL-10 and TGF-β, inhibit the pro-inflammatory activity of M1-type macrophages, and reduce the release of pro-inflammatory factors. Bouchareychas et al. [211] found that exosomes from IL-4-polarized mouse bone marrow-derived macrophages (BMDM-IL-4-exo) are rich in miR-99a/146b/378a, which exert anti-inflammatory effects by inhibiting the TNF-α/NF-κB signaling pathway. In the ApoE−/− mouse model, BMDM-IL-4-exo significantly reduced bone marrow multipotent progenitor cells and circulating monocytes, and decreased plaque macrophage infiltration by 45%. Wang et al. [212] demonstrated that M2 macrophage-derived exosomes (ExoM2) significantly inhibited PDGF-BB-induced proliferation and migration of vascular smooth muscle cells (VSMCs) and reversed their pathological transformation from a contractile phenotype to a synthetic phenotype. In the ApoE−/− mouse model, after 4 weeks of ExoM2 treatment, the plaque area decreased by 41%, the necrotic core shrank by 53%, and the expression of MMP9 in plaques decreased, indicating significantly enhanced plaque stability. In general, EVs secreted by M2-type macrophages play a protective role in the treatment of atherosclerosis: by anti-inflammation, promoting lipid clearance, enhancing cell burial and stabilizing the fibrous cap of plaques, they delay the progression of the disease. However, the in vitro polarization conditions of M2-type macrophages need to be optimized, and more research is needed for further verifying the long-term safety and therapeutic effect of EV derived from M2 macrophage.

##### EPCs

EPCs, the precursor cells of vascular endothelial cells, originate from the bone marrow and are also present in small quantities in peripheral blood, umbilical cord blood, and adipose tissue [201]. These cells can migrate to injury or ischemic sites, differentiating into endothelial cells and contributing to angiogenesis and repair processes. Statins and exercise can increase their quantity and function, while risk factors for coronary heart disease such as diabetes and smoking can reduce their quantity and activity. EPC-based therapy is expected to cure the vascular diseases of patients with AS. EPCs can nest to damaged endothelial sites, promote re-endothelialization, maintain endothelial integrity, and reduce the risk of atherosclerosis and thrombosis. Kong et al. [213] observed in a carotid artery balloon injury model that EPC-derived exosomes increased the re-endothelialization area by 80% within 14 days by accelerating endothelial cell migration and inhibited excessive proliferation of smooth muscle cells (SMCs) through paracrine effects. Bai et al. [214] found that EPCs-derived exosomes are rich in AS-related miRNAs such as miR-21a-5p and miR-222-3p, which significantly improved endothelium-dependent vasodilation function and reduced aortic plaque area in diabetic AS mice by inhibiting the secretion of inflammatory factors such as TNF-α and IL-8. Similarly, Li et al. [215] reported that EPCs-derived exosomes deliver miR-199a-3p to endothelial cells, inhibiting ferroptosis by targeting the SP1 protein, thereby reducing lipid peroxidation and endothelial damage. This study confirmed in the ApoE−/− mouse model that EPCs-derived exosomes significantly reduced ROS levels in aortic plaques and serum concentrations of IL-6 and TNF-α. In general, EPCs-derived EVs can maintain endothelial integrity and inhibit the inflammatory, which is benefit for AS treatment. However, the isolation techniques and classification and origin of EPCs is ambiguous, which requires more preclinical research regarding the biology of EPCs, culture assays, surface markers, origin, and differentiation hierarchy.

##### EVs Generated from Cells Under Stimulation

In addition to directly isolating EVs from different cell sources for AS treatment, some studies have investigated the therapeutic effects of EVs generated from cell sources under various stimulations. It has been reported that pretreatment with cytokines, drugs, hypoxia, or physical factors can improve the biological functions of donor cells, and prepare them with the desired characteristics [216]. For example, baicalin (Ba), the main active component of Scutellaria baicalensis, has anti-inflammatory and antioxidant properties. Researchers studied the therapeutic effects of exosomes produced by MSCs pretreated with Ba (Ba-exos). Ba-exos reduced serum levels of inflammatory factors more effectively than MSC-exos without Ba treatment [217]. In addition to drug stimulation, vesicles generated by physical stimulation represent another approach. For example, a study aimed to explore the regulatory effect of EVs derived from endothelial cells stimulated with laminar shear stress (LSS-EVs) on macrophage polarization and their anti-atherosclerotic potential [218]. Researchers stimulated HUVECs with simulated laminar shear stress (15 dyn/cm^2^) in vitro and isolated LSS-EVs by ultracentrifugation. miRNA-seq showed that miR-34c-5p was significantly upregulated in LSS-EVs. LSS-EVs reshaped macrophage polarization by upregulating miR-34c-5p, promoting the transformation of M1 to M2 macrophages. In general, under the action of specific exogenous stimuli, donor cells can activate the changes in membrane proteins and EVs’ contents, thereby obtaining EVs with ideal anti-atherosclerotic activity.

**Table 2:** Examples of EVs from specific cell sources for therapeutic applications.

The Source of EVs	The Major Cargo	Target Pathway	The Influence on AS	Ref.
MSCs	FENDRR	FENDRR targeted miR-28 to increase TEAD1 activation	Reducing HUVEC-C injury and atherosclerotic plaque formation.	[219]
MSCs	miR-146a	miR-146a could suppress Src phosphorylation and downstream targets VE cadherin and Caveolin-1.	MSC-sEV mitigated endothelial cell senescence and stimulate angiogenesis.	[206]
MSCs	miR-21a-5p	Targeted inhibition of the KLF6 and ERK1/2 pathways	Promote the polarization of macrophages to type M2 and reduce their migration, thereby alleviating the formation of AS plaques and inflammatory responses.	[207]
MSCs	miR-145	Inhibit the expression of JAM-A	Reduce endothelial migration and barrier disruption, thereby inhibiting plaque formation.	[220]
MSCs	miR-let7	Inhibit the HMGA2/NF-κB pathway and down-regulate the IGF2BP1/PTEN pathway	Regulating the phenotype of macrophages alleviates the progression of AS.	[221]
BMSCs	long non-coding RNA AU020206	Block CEBPB-mediated transcriptional activation of NLRP3	Regulating the phenotype of macrophages alleviates the progression of AS.	[222]
BMSCs	-	Inhibiting NLRP3/Caspase-1/GSDMD in the pyroptosis pathway	Alleviate atherosclerosis by regulating the pyroptosis pathway and metabolic/inflammation-related genes.	[223]
BMSCs	-	Up-regulate phosphorylated AMPKα and inhibit mTOR activation	Regulating autophagy and polarization of macrophages alleviates diabetic AS.	[224]
M2 polarization of naive BMDMs induced by IL-4	microRNA-99a/146b/378a	Inhibiting NF-kB and TNF-a signaling pathways	Inhibit inflammatory signals, regulate the hematopoietic process, and significantly improve the stability of atherosclerotic plaques.	[211]
ADSCs	miR-26	The upregulation of miR-26 can reduce the mRNA expressions of TNF-α, IL-6 and IL-1β	miR-26 inhibits the progression of carotid atherosclerosis by regulating lipid metabolism and inflammatory responses.	[225]
hiPSCs	miR-126	miR-126 inhibits the PI3K/Akt/mTOR pathway	Activate endothelial autophagy and effectively alleviate arterial stenosis induced by inflammatory injury.	[199]
hUCMSC	miR-100-5p	Target FZD5 and inhibit the Wnt/β-catenin pathway	Inhibit eosinophil migration, promote their apoptosis, and alleviate inflammatory responses.	[226]
Human fetal aorta-derived EPCs	-	-	Inhibit the formation of new intima after carotid artery injury by promoting endothelial repair.	[213]
EPCs	miR-199a-3p	miR-199a-3p reduces the expression of SP1 and upregulates the antioxidant proteins SLC7A11 and GPX4	Reduce ROS, lipid peroxidation and iron accumulation, thereby reducing endothelial cell ferroptosis and alleviating the progression of AS.	[215]
RBCs	heme	Activate the HO-1 pathway by delivering heme	Regulate the transformation of macrophages to anti-inflammatory phenotypes and inhibit the formation of foam cells.	[227]
Efferocytes	prosaposin	Activate the GPR37 receptor of macrophages and up-regulate the expression of Tim4	Enhance the ability of efferocytes macrophages to continuously clear apoptotic cells.	[202]
M2 polarization of RAW264.7 macrophages induced by IL-4	-	-	Inhibit the proliferation, migration and synthetic phenotypic transformation of VSMCs, significantly delay the progression of AS and enhance the stability of plaques.	[212]
Baicalin-pretreated MSCs	-	Up-regulate SIRT1 and inhibit the activation of the NF-κB pathway	Reduce the inflammatory response and plaque formation of VSMCs	[217]
Endothelial cells sheared by laminar flow	miR-34c-5p	miR-34c-5p inhibits TGIF2, activates the nuclear translocation of Smad3, and promotes the TGF-β signaling pathway	Regulating the M2 polarization of macrophages to achieve anti-atherosclerotic treatment	[218]

Abbreviations: MSCs, Mesenchymal Stem Cells; BMSCs, Bone marrow stem cells; BMDMs, naive bone marrow-derived macrophages; ADSCs, Adipose-derived stem cells; hiPSCs, Human-induced pluripotent stem cells; hUCMSC, Human umbilical cord mesenchymal stem cells; EPCs, Endothelial progenitor cells; RBCs, Red blood cells; FENDRR, fetal-lethal non-coding developmental regulatory RNA; Tim4, T-cell immunoglobulin domain; TNF-α, Tumor Necrosis Factor-α; IL-6, Interleukin-6; IL-1β, Interleukin-1β; PI3K, Phosphatidylinositol 3-Kinase; Akt, Protein Kinase B; mTOR, Mammalian Target of Rapamycin; FZD5, frizzled 5; SP1, specificity protein 1; SLC7A11, Solute Carrier Family 7 Member 11; GPX4, Glutathione Peroxidase 4; HO-1, heme oxygenase-1; SIRT1, sirtuin 1; TGIF2, TG-interacting factor.

#### 3.3.2. Drug-Loaded EVs

The contents of EVs are affected by factors such as the culture conditions of donor cells and the extraction process. Loading biological cargo into EVs offers a viable approach for significantly increasing therapeutic efficacy. The drug-loading strategies for EVs are primarily divided into two categories: exogenous and endogenous (Figure 4B). Exogenous drug delivery involves extracting and purifying EVs, and then encapsulating therapeutic drugs using methods such as coincubation [228], electroporation [229], extrusion [230], freeze–thawing [231], sonication [232], and surfactant treatment [233]. Exogenous approach offers relatively simple operation and high drug-loading efficiency, but may compromise EVs’ integrity during loading and requires additional purification to remove unencapsulated drugs [234]. Endogenous drug delivery introduces target molecules (e.g., nucleic acids, viral proteins, chemical drugs) into donor cells via genetic engineering or co-incubation, allowing them to be actively or passively sorted into EVs during cytoplasmic processing [235,236]. The drug-loaded EVs are then isolated and purified. This method preserves EVs’ integrity and functionality by avoiding direct manipulation of EVs, but it is necessary to consider that drugs loaded by incubation should have a low toxicity for parental cells [237]. Both strategies have distinct advantages and limitations, requiring selection based on drug characteristics in practical experiments.

##### Gene Drugs

Gene therapy can design targeted strategies against core pathological mechanisms of AS (e.g., lipid metabolism disorders, inflammatory responses, endothelial dysfunction) for precise treatment. However, naked gene drugs are highly susceptible to nuclease degradation in the bloodstream, which could be solved by using EVs as a promising new delivery platform for gene delivery [238]. Transfection is a common method to encapsulate gene drugs into EVs. Host cells are genetically engineered to carry plasmids of target genes, enabling intracellular overexpression and active packaging of gene drugs into exosomes [235]. miR-145 is the most abundant miRNA in arteries and a key regulator of the VSMC phenotype. Its expression is basically downregulated in the arteries of atherosclerotic mice and humans. Yang et al. [220] overexpressed miR-145 in MSCs using lentiviral transfection, and then isolated miR-145-enriched exosomes. These MSC-derived miR-145-enriched exosomes regulated endothelial cell function and reduced atherosclerotic plaques by targeting and inhibiting JAM-A. Another advantage of artificially loading genes into exosomes lies in the ability to design genes in the form of prodrugs. Interleukin-10 (IL-10), a cytokine critical for limiting inflammation and preventing tissue damage, can inhibit AS progression when delivered therapeutically. However, systemic administration is restricted by side effects. Bu et al. [239] engineered IL-10 mRNA (IRES-IL-10 mRNA) to depend on an inflammatory marker for translation. They overexpressed IRES-IL-10 mRNA in HEK293T cells using plasmid transfection, and then isolated IRES-IL-10 mRNA-enriched exosomes (ExoIRES-IL-10). This system effectively alleviated AS symptoms in ApoE−/− mice with minimal side effects. Further development of highly efficient and low-toxicity gene transfection vectors (such as non-viral vectors) is still needed to enhance the therapeutic effect.

##### Chemical Drugs

Traditional chemical drugs for AS (e.g., statins, aspirin) may cause risks of systemic toxicity with long-term use [17]. EVs offer natural targeting, biocompatibility, low immunogenicity, and prolonged circulation, making them ideal for enhancing efficacy and reducing toxicity. Common drug-loading methods include hydrophobic interaction and electroporation. Electroporation is based on the fluid mosaic model of cell membranes, and this technique uses an electric field to reorient phospholipid polar head groups, forming nanoscale pores [240]. Drugs enter EVs via electrophoresis and concentration gradient diffusion. Electroporation can offer high drug-loading efficiency. For instance, electroporation was used to load FDA-approved 5-aminolevulinic acid hexyl ester hydrochloride (HAL) into M2-derived exosomes (HAL@M2 Exo), achieving a loading efficiency of 25.14% and encapsulation efficiency of 19.34% [241]. Targeted delivery HAL via HAL@M2 Exo reduced aortic plaque area by 75.2%. However, high-voltage pulses may cause irreversible damage to exosomes, leading to aggregation, fusion, altered membrane potential, reduced colloidal stability, and EVs degradation [242]. In another study, a platelet-derived EV (PEV)-based targeting system loaded an anti-inflammatory drug (MCC950) via hydrophobic interaction [243]. MCC950-PEVs achieved a drug-loading rate of 7.1% by the hyd rophobic interaction method, successfully delivering MCC950 to atherosclerotic plaques, inhibiting inflammation, reducing plaque size, and minimizing systemic side effects. The method of hydrophobic incubation has a relatively low drug load, but it can maintain the integrity of EVs. Therefore, it is necessary to develop a method that can both maintain the integrity of the EVs and achieve high levels of drug loading.

#### 3.3.3. EV Mimics

Natural exosomes need to be extracted from cell culture media or biological fluids (such as blood and urine), with extremely low yields and complex purification processes, resulting in high costs and making it difficult to meet clinical or industrial demands. Exosome mimics can be prepared through artificial synthesis without relying on natural secretion processes, enabling large-scale production, significantly reducing costs and making it easier to promote industrial application (Figure 4C). According to the International Society for Extracellular Vesicles (ISEV), EV mimetic refers to EV-like particles produced through direct artificial manipulation [173].

Artificial cell-derived vesicles (ACDVs) are EV mimetics generated in laboratories via induced cell disruption (e.g., extrusion) [173]. Mimetic vesicles can also be developed by fusing multiple cell types to enhance targeting efficiency [244,245]. This includes generating cell-derived nanovesicles (CDN) by rupturing production cells, thereby producing vesicles with EV-like size and biological functions, while being produced at a relatively high yield. The common source cell types of CDN include red blood cells, immune cells, cancer cells, mesenchymal stem cells and normal tissue cells. CDN is typically produced through extrusion, ultrasonic treatment, hypoosmotic treatment, nitrogen cavitation, repeated freeze–thaw cycles, cell homogenizers, or a combination of these methods. CDN can effectively load therapeutic proteins, small molecules or nucleic acids and be engineered to modify surface proteins. Ko et al. [196] efficiently produced exosome-mimetic nanovesicles (FT/NVs) from umbilical cord mesenchymal stem cells using a freeze–thaw treatment combined with a tangential flow filtration system, with a yield approximately 3 times higher than that of traditional methods. These FT/NVs share similar characteristics with traditional nanovesicles and can effectively inhibit tumor necrosis factor-α-induced inflammation in endothelial cells. Although CDN is a promising alternative to natural EVs for drug delivery, a comprehensive toxicity assessment must be conducted before clinical translation. Synthetic vesicles (SVs) are EV mimetics synthesized de novo from molecular components or constructed as hybrid entities (e.g., fusions of liposomes and native EVs) [173,246]. By integrating functional proteins into the phospholipid membrane of liposomes, protein-functionalized liposomes (PFLs) with exosome-like vesicle properties can be designed. Researchers incorporated three exosomal components related to transport (anionic lipids, cholesterol, and aquaporin-1) into PFLs. Compared with traditional lipid nanoparticles, this significantly increased their effective diffusion coefficient in biological tissues, which is expected to provide an optimization direction for the design of highly permeable lipid nanoparticles [247]. However, the effectiveness of PFLs in the treatment of atherosclerosis still needs to be verified through extensive preclinical studies.

#### 3.3.4. Engineering Strategies for EVs and EV Mimics

The key challenges in EV heterogeneity, yield and purity of EV separation have hindered the standardized production of clinical-grade EV therapies. In addition, the targeting efficiency of EVs is highly influenced by parental and target cells. Meanwhile, EVs exhibit complex compositions, with their biodistribution affected by multiple factors, resulting in indistinct tissue specificity. These vesicles could be rapidly filtered by the liver and kidney tissue [248]. Compared to natural EVs, an increasing number of preclinical studies have begun to develop engineered vesicles to enhance biocompatibility, adjustability and scalability. Engineered nanovesicles can acquire additional functions (e.g., targeting) through fusion with cell membranes or liposomes.

Engineered nanovesicles can be produced by fusing the cell membrane with EVs. This method can preserve the therapeutic activity of EVs while enhancing their targeting ability. For example, in order to enhance the targeting ability of EVs, a number of studies fused EVs with platelet membranes to create engineered nanovesicles. M2-derived EVs (M2EVs) exhibit anti-inflammatory and tissue-repair properties but suffer from poor in vivo targeting. Platelet membrane surface proteins (e.g., GPIb) can target macrophages in atherosclerotic plaques. By developing platelet membrane-decorated M2EVs (P-M2EVs), the study combines platelet targeting with M2EV therapeutic functions for precise AS treatment [249]. M2EVs and platelet membrane vesicles (PMVs) were fused at a 1:1 ratio via sonication and extrusion, achieving an 87.17% membrane fusion efficiency. Western blot confirmed the retention of EV marker CD63 and platelet marker GPIb after fusion. In vivo targeting showed that P-M2EVs exhibited 3.35-fold higher fluorescence intensity in the aorta compared to M2EVs, with enhanced penetration into plaque cores. The P-M2EV group showed a 45% reduction in aortic plaque area, a 1.8-fold increase in collagen content, and a 60% reduction in macrophage infiltration compared to the control group. In another study, an anti-inflammatory delivery system targeting plaque macrophages was developed by combining the natural anti-inflammatory properties of mesenchymal stem cell-derived EVs (MSC-EVs) with platelet membrane modification for enhanced targeting [250]. After intravenous injection, P-EVs exhibited 1.5-fold higher fluorescence intensity in aortic plaques and reduced liver accumulation compared to unmodified EVs. In general, compared with EVs, engineered nanovesicles have a higher yield and the potential to enhance therapeutic effects.

Another kind of engineered nanovesicles can be produced by fusing liposomes with EVs. There are various strategies for the fusion of liposomes and exosomes, such as co-incubation and repeated freeze–thaw cycles. This approach, which involves fusing natural exosomes with liposomes to form larger and more functional engineered exosome/liposome hybrid nanovesicles, significantly improves drug loading efficiency and AS targeting ability [251]. Phospholipid materials used for preparing liposomes are mainly divided into two categories: natural phospholipids and synthetic phospholipids. The natural phospholipids include phosphatidylcholine, phosphatidylethanolamine, phosphatidylserine, phosphatidylglycerol and cardiolipin. The synthetic phospholipids mainly including dipalmitoylphosphatidylcholine, distearoylphosphatidylcholine and dimyristoylphosphatidylcholine. These diverse phospholipid materials can provide more functions for nanocarriers, such as cation characteristics, temperature sensitivity and pH responsiveness. Zheng et al. [252] developed a novel engineered nanorobot UM-EVLipo by integrating a nanomotor into hybrid nanovesicles. EVs secreted by IL-4-stimulated bone marrow macrophages (BMDM) were fused with SHP-1 siRNA-loaded liposomes via liquid nitrogen freeze–thawing to form EV-Lipo heterovesicles. Urease motors were precisely decorated on one side of the vesicles to create UM-EVLipo. Urease catalyzes the decomposition of blood urea into CO_2_ and NH_3_, generating microbubbles to propel directional nanoparticle movement. Urease-driven propulsion enables rapid endothelial penetration and shortens targeting time. Gas production from urea decomposition induces mechanical stress on lysosomal membranes, promoting vesicle escape into the cytoplasm and protecting siRNA from degradation. In general, this method utilizes liposome membranes for simple surface modification and effectively avoids the defect of liposomes lacking endogenous functions. Furthermore, the design of SVs provides EVs more functions to enhance the therapeutic effect.

Although the exosome/liposome hybrid nanovesicles demonstrate an outstanding therapeutic effect, the acquisition process of EVs is complex, and the yield is relatively low. The hybrid vesicles prepared by fusing cell membranes and liposomes may have a higher yield and they are also multi-functional. For example, Qiu et al. [253] developed hybrid membrane nanovesicles (HMNVs) by fusing macrophage membranes overexpressing MerTK with HEK293T cell membranes overexpressing transferrin receptor (TfR), and integrating DOPE polymers and superparamagnetic iron oxide nanoparticles (SMNs). The TfR-mediated transferrin binding capacity synergized with the magnetic navigation of SMN to significantly enhance nanoparticle accumulation in aortic plaques, increasing drug concentration in the plaque area by 3.2-fold. DOPE promoted fusion between HMNVs and recipient macrophage membranes, directly replenishing MerTK protein to restore apoptotic cell clearance in macrophages of diabetic mice, with a 68% improvement in efferocytosis efficiency and effective reversal of diabetes-related AS progression. In general, engineered nanovesicles prepared by fusing cell membranes and liposomes are with higher yield and can provide researchers with more space for design.

## 4. Prospects for Clinical Translation

This review classifies the applications of organic nanoparticles in AS treatment through bibliometric analysis, highlighting three primary research directions including vascular organic stents, traditional polymeric nanoparticles, and biomimetic organic nanoparticles. Vascular organic stents have been widely used in the interventional treatment of coronary heart disease after 40 years of development. The Swiss team led by Urich Sigwart pioneered the development of bare metal stents (BMS), which provide mechanical vascular support through Percutaneous Transluminal Coronary Angioplasty (PTCA), thereby reducing the rate of postoperative restenosis. Subsequently, drug-eluting stents (DES) composed of organic polymeric materials such as PLLA, PLGA were developed to inhibit intimal hyperplasia through local sustained release of antiproliferative drugs, further reducing the restenosis rate [254]. Among them, the Orsiro stent and SYNERGY stent have demonstrated excellent preliminary efficacy and safety data in clinical evaluations. Future research aims to improve patient compliance by modifying the types of organic polymeric materials to reduce inflammatory responses after stent implantation and developing next-generation DES that selectively inhibit smooth muscle cells proliferation while sparing vascular endothelial cells.

Traditional nanoparticles such as liposomes have emerged as attractive drug delivery systems for AS treatment due to their capacity for targeted delivery. Engineering these nanoparticles to respond to pathological stimuli in the vascular microenvironment may enable in situ cargo release at target sites and improve the bioavailability of therapeutic agents. In the field of AS treatment, more and more clinical research on applying traditional organic polymeric nanoparticles for AS treatment continues. For example, CSL112 is a lipoprotein particle composed of human plasma apoA-I and soybean phosphatidylcholine, which promotes cholesterol efflux via the ABCA1 pathway to inhibit AS progression. The AEGIS-I clinical trial (NCT02108262) confirmed that CSL112 significantly enhances cholesterol efflux capacity [255]. However, the subsequent phase III trial (NCT03473223) showed no significant reduction in the incidence of primary endpoints. Nevertheless, patients receiving CSL112 infusions exhibited a trend toward reduced cardiovascular mortality and myocardial infarction (including type 1 myocardial infarction and stent thrombosis-related myocardial infarction) compared to the placebo group [256]. These clinical results indicate that nanodrugs exhibit therapeutic effects in a small sample size, but their efficacy diminishes with larger sample sizes, highlighting ongoing challenges in the clinical translation of traditional organic nanodrug delivery systems. Curcumin nanomicelles can prevent the progression of atherosclerosis by reducing the level of the inflammatory marker high-sensitivity C-reactive protein (IRCT20130811014330N4). Further large-scale and multicenter clinical trial studies appear to be helpful in validating its therapeutic effects. Whether active targeting or smart-responsive nanoparticles can overcome this dilemma remains a promising area of exploration.

The application of organic biomimetic nanoparticles such as EVs in AS treatment represents the most cutting-edge and rapidly evolving research direction. Compared to traditional nanoparticles, EVs exhibit lower immunogenicity, minimal risk of immune rejection, and higher safety. However, their clinical translation is still in the early stages, with no EV-based therapies currently approved for market use. Key obstacles include challenges in large-scale production technical hurdles, the lack of unified quality standards, and concerns over the high production costs. Whether EV-based nanomedicines can offer a cost-effective and scalable solution to AS treatment remains to be determined. The development of engineered EVs with improved therapeutic effect may offer a promise for overcoming these barriers.

Research on nanomedicines relies on reproducible and scalable production technologies, with benefits and risks balanced through Good Manufacturing Practice (GMP) and clinical trials. GMP is a key component of the quality assurance (QA) system for nanomedicines, aiming to ensure that nanomedicines are continuously and stably produced in accordance with regulatory requirements through reproducible and scalable manufacturing processes, thereby guaranteeing consistent quality. Due to the unique size (1–100 nm), matrix composition, and surface properties of nanomedicines, the complexity and specificity of their production processes impose higher requirements on GMP. During the scale-up from small-scale laboratory production to large-scale industrial manufacturing, GMP is essential to maintain the stability of key properties such as physicochemical characteristics and pharmacokinetics, while reducing toxicological risks. For liposomal nanomedicines, emphasis should be placed on stability, pharmacokinetics, and control of production process deviations. For block copolymer micelle-based nanomedicines, it is necessary to standardize chemical manufacturing and control (CMC), non-clinical toxicology, and early clinical trial design. For surface-coated (e.g., PEGylated) nanomedicines, the impact of the coating on plasma circulation time and stability needs to be evaluated to ensure detailed and accurate product descriptions.

## Supplementary Materials

The following supporting information can be downloaded at: https://www.mdpi.com/article/10.3390/pharmaceutics17091131/s1. Figure S1. (A) Co-occurrence analysis of keywords with at least 15 occurrences. Node size indicates the occurrence frequency; node color represents the cluster. (B) Overlay visualization of terms based on the average publication year.
