# Supplementary figures and images for: A Bibliometric Analysis of Strategies for Atherosclerosis Treatment with Organic Nanoparticles
Source: Pharmaceutics. 2025 Aug 29;17(9):1131. doi: 10.3390/pharmaceutics17091131 (PMC12473198; doi:10.3390/pharmaceutics17091131)

Jizhuang Ma, Xia Zhao, Xinwen Xu, Lixin A, Qiang Liu \* and Peng Qu

\* Correspondence: andyliu\_1844@dlut.edu.cn

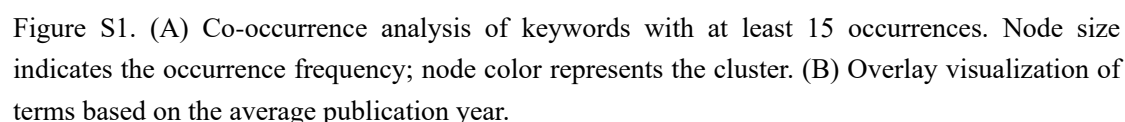

Supplement: Supplementary file 1 [file pharmaceutics-17-01131-s001.zip › pharmaceutics-3801055-supplementary.pdf]
